# Loss of sister kinetochore co-orientation and peri-centromeric cohesin protection after meiosis I depends on cleavage of centromeric REC8

**DOI:** 10.1016/j.devcel.2021.10.017

**Published:** 2021-11-22

**Authors:** Sugako Ogushi, Ahmed Rattani, Jonathan Godwin, Jean Metson, Lothar Schermelleh, Kim Nasmyth

**Affiliations:** 1 Department of Biochemistry, University of Oxford, Oxford OX1 3QU, UK; 2 The Hakubi Center for Advanced Research, Kyoto University, Kyoto 606-8501, Japan

**Keywords:** meiosis, oocyte, cohesin, kinetochore orientation, Separase, cohesin protection, shugoshin, mouse, mammal

## Abstract

Protection of peri-centromeric (periCEN) REC8 cohesin from Separase and sister kinetochore (KT) attachment to microtubules emanating from the same spindle pole (co-orientation) ensures that sister chromatids remain associated after meiosis I. Both features are lost during meiosis II, resulting in sister chromatid disjunction and the production of haploid gametes. By transferring spindle-chromosome complexes (SCCs) between meiosis I and II in mouse oocytes, we discovered that both sister KT co-orientation and periCEN cohesin protection depend on the SCC, and not the cytoplasm. Moreover, the catalytic activity of Separase at meiosis I is necessary not only for converting KTs from a co- to a bi-oriented state but also for deprotection of periCEN cohesion, and cleavage of REC8 may be the key event. Crucially, selective cleavage of REC8 in the vicinity of KTs is sufficient to destroy co-orientation in univalent chromosomes, albeit not in bivalents where resolution of chiasmata may also be required

## Highlights

• The meiotic segregation pattern is dictated by properties built in chromosomes
• Catalytic activity of Separase is vital for transformation of bivalents into dyads
• SGOL2 controls cohesin kinetics during the meiosis I/II transition
• Centromeric cohesin ensures meiotic kinetochore co-orientation and cohesin protection

## Graphical abstract

**Figure:**
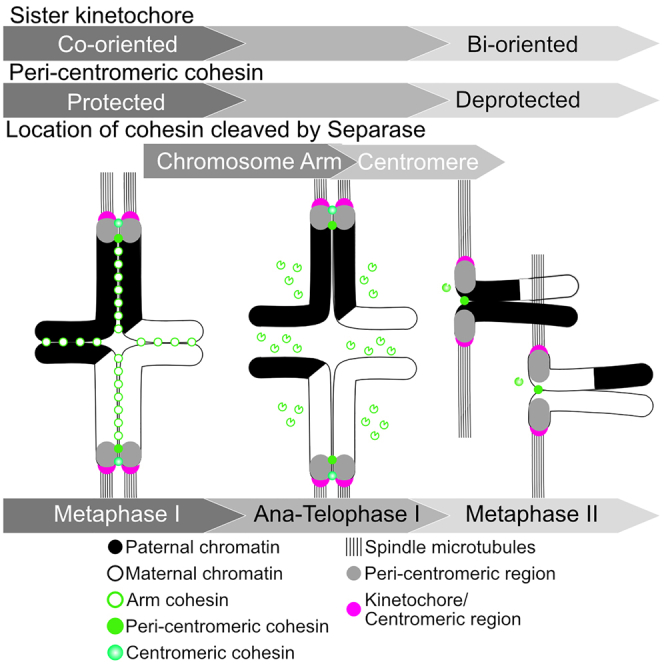

## Teaser

During meiosis I, peri-centromeric cohesion and the attachment of sister kinetochores to microtubules emanating from the same pole ensure that sister chromatid dyads remain associated. Ogushi et al. discover that cohesin cleavage by Separase converts kinetochore orientation and de-protects peri-centromeric cohesion to enable dyad resolution and segregation during meiosis II.

## Introduction

Production during meiosis of haploid gametes from diploid germ cells is only possible because two rounds of chromosome segregation occur without an intervening round of DNA replication. Meiotic DNA replication is accompanied by establishment of cohesion between sister DNAs and recombination between homologous non-sister DNAs, creating chiasmata, joins all four homologous chromatids together and forms bivalents (Nasmyth, 2015; Watanabe, 2012).

During meiosis I, sister kinetochores (KTs) act as a single unit (co-orientation). Consequently, pairs of maternal and paternal KTs, but not sister KTs, are pulled by microtubules. The first division is triggered by cleavage of meiotic cohesin’s REC8 along chromosome arms by a thiol protease ESPL1 called Separase, which resolves chiasmata and creates dyads (Kudo et al., 2006). Two chromatids of these dyads are held together through cohesion between their peri-centromeres, and sister KTs are pulled in opposite directions (bi-orientation) until Separase cleaves remaining cohesin molecules during the second meiotic division. Therefore, two key features distinguish the first meiotic division from the second and from mitosis, namely, co-orientation of sister KTs and protection of peri-centromeric (periCEN) cohesin. How these are conferred and how they are both subsequently lost, so that during meiosis II sister KTs are bi-oriented and Separase cleaves periCEN cohesin thereby converting dyads into chromatids, is poorly understood.

Common to most species, periCEN cohesin is protected from Separase during meiosis I by the Shugoshin/Mei-S332 (Sgo) protein family, which forms a highly conserved homodimeric parallel coiled-coil that binds protein phosphatase 2A, PP2A-B56 (Kitajima et al., 2006; Riedel et al., 2006; Xu et al., 2009). This protection arises because REC8’s prior phosphorylation, which is required for cohesin cleavage by Saparase, is presumably removed by PP2A recruited by Sgo (Brar et al., 2006; Katis et al., 2010). What is less clear is that how Sgo ensures PP2A de-phosphorylation selectively within periCen REC8. Equally mysterious is the mechanism by which protection of periCEN cohesin from Separase is lost by the time cells initiate anaphase II (AII). In budding yeast, degradation by the anaphase-promoting complex/cyclosome (APC/C) of its sole Sgo ortholog, Sgo1, at AII onset ensures that PP2A is removed simultaneous with Separase activation (Jonak et al., 2017). However, SGOL2, which confers protection during meiosis I in mammals, does not share this property (Marston, 2015) and it has instead been suggested that protection is destroyed by tension created within periCEN chromatin via bi-orientation of sister KTs during meiosis II (Lee et al., 2008). This proposal provides no explanation for how such tension ablates Sgo’s activity nor why bi-orientation of sister KTs in yeast monopolin mutants is not accompanied by deprotection (Tóth et al., 2000). The role of other factors proposed to have a role in deprotection, such as I2PP2A and cyclin A, remains unclear (Touati et al., 2012; Wassmann, 2013).

Unlike protection of centromeric (CEN) cohesin, the mechanism responsible for sister KT co-orientation during meiosis I appears to differ among species (McKinley and Cheeseman, 2016). In budding yeast whose point centromeres contain a single CENPA nucleosome, co-orientation during meiosis I depends on a monopolin complex, the core of which is the V-shaped Csm1 heterodimer, that is thought to confer co-orientation by cross-linking sister KTs (Corbett and Harrison, 2012; Rabitsch et al., 2003). Because Csm1 has little role in co-orientation in fission yeast *S*.*pombe* (Gregan et al., 2008) and is absent from metazoan genomes (Plowman et al., 2019), some other mechanism must confer co-orientation in eukaryotes with regional centromeres. In fission yeast, a meiosis cohesin containing Rec8 is necessary to prevent bi-orientation during meiosis I (Watanabe and Nurse, 1999; Sakuno et al, 2009). This suggests that cohesin mediates co-orientation by holding sister KTs together. Although plausible, this hypothesis has never been rigorously tested. In an attempt to cleave specifically the CEN cohesin using fission yeast strains expressing a CenpC-TEV and Rec8 containing TEV sites, centromere-specific depletion of cohesin was not clearly shown (Yokobayashi and Watanabe, 2005).

Another key meiotic regulatory factor in both fungi and metazoa is a family of proteins related to Spo13 in budding yeast (Wang et al., 1987), Moa1 in fission yeast (Yokobayashi and Watanabe, 2005), and Meikin (Kim et al., 2015) in mammals. Although not highly conserved in amino-acid sequences, all members of the family are expressed exclusively during meiosis and have the property of recruiting Polo-like kinases (PLK) to KTs. Because their ablation compromises protection of periCEN cohesion by Sgo and regulation of the APC/C activity, as well as co-orientation, these Spo13-like proteins should be viewed as factors that regulate numerous properties of meiotic KTs (Katis et al., 2004; Kim et al., 2015; Klapholz and Esposito, 1980; Lee et al., 2004; Shonn et al., 2002).

To address the mechanisms conferring co-orientation and periCEN protection in mouse oocytes, we have adopted a technique developed more than 40 years ago with grasshopper spermatocytes, namely, the transfer of chromosomes and their spindles from meiosis I to meiosis II cells and vice versa (Nicklas, 1977). Our findings confirm that both co-orientation of sister KTs and periCEN cohesin protection are conferred by the state of chromosomes and their spindles and not by the nature of cytoplasm. By combining cytological and genetic manipulations, we show that Separase cleavage activity converts chromosomes from a meiosis I to a meiosis II state, most likely through cleavage of CEN REC8-containing cohesin. Our findings suggest that CEN cohesin not only confers co-orientation of sister KTs, but more unexpectedly, also helps Sgo protect from Separase periCEN cohesin located many megabases away (Vissel and Choo, 1989). CEN cohesin is also protected by SGOL2 upon Separase activation during meiosis I. However, unlike periCEN cohesin, which survives until metaphase II (MII), the protection of CEN cohesin only lasts until late telophase I (TI), whereupon REC8 cleavage induces sister KTs to split into their component parts, setting the scene for their subsequent bi-orientation during MII.

## Results

### Dyads retain their MII character when transferred to metaphase I (MI) oocytes

**Figure 1:**
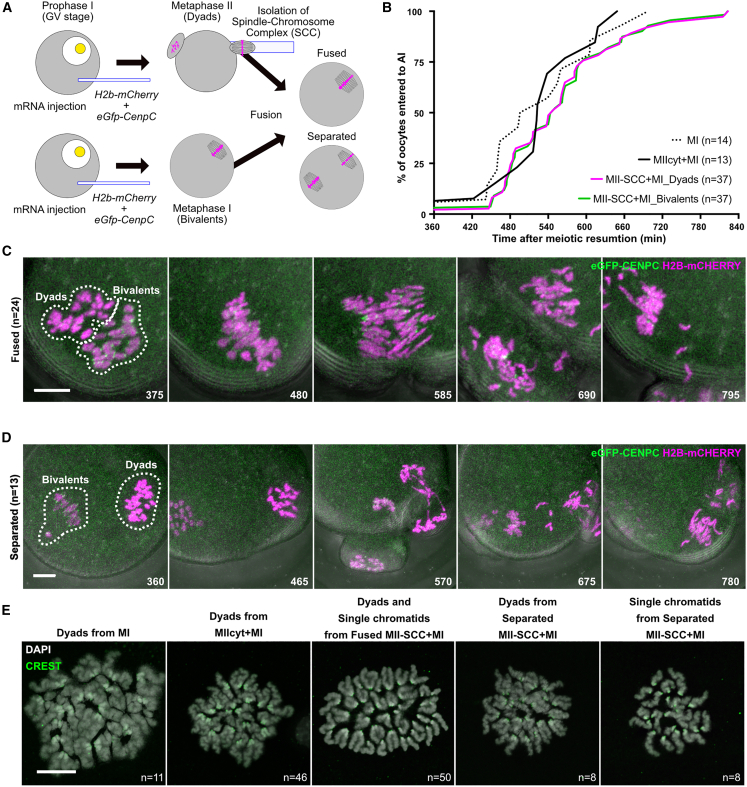
Either sister KT bi-orientation or deprotection of cohesion is conferred by the nature of dyads itself rather than the surrounding factors coming from cytoplasm (A) Schematic of an experiment showing that a SCC containing dyads from an oocyte at MII is fused with an oocyte at MI, and a resulting oocyte is called MII-SCC+MI. (B) Segregation timing of chromosomes in MII-SCC+MI. (C and D) Live cell imaging of chromosome segregation in MII-SCC+MI, whose MII-SCC was fused (C) or not fused with a host MI spindle (D). Numbers indicate the time after meiotic resumption (min). H2B-mCherry (magenta), chromosomes. eGFP-CENPC (green), KTs. Bars, 10 μm. (E) Single chromatid formation from transferred dyads after the first meiotic division (CREST: green; DAPI: gray). Bar, 15 μm. n, the numbers of oocytes analyzed.

To address whether factors associated with the spindle-chromosome complex (SCC) or those within the cytoplasm confer co-orientation and/or periCEN cohesion protection, we used microsurgery to transfer SCCs between oocytes. We first addressed how dyads isolated from MII oocytes (donors) behave when placed inside MI oocytes (hosts). SCCs from MII oocytes, which are surrounded by membrane, were placed in contact with MI oocytes and fused with them using envelopes from Hemagglutinating Virus of Japan (HVJ-E) (Figure 1A). The overall fusion success rate was 95% (1,014/1,067). Chromosome behavior was observed by injection of mRNAs, *H2b-mCherry* (chromosomes), and *eGfp-CenpC* (KTs). To assess the effect of cytoplasm transferred along with MII SCCs, we fused MI oocytes with cytoplasts from MII oocytes with a similar volume to SCCs (MIIcyt+MI). Notably, neither the frequency nor timing of chromosome segregation differed substantially between intact unmanipulated oocytes (86%, 14/16, 534 ± 79 min), MIIcyt+MI oocytes (76%, 13/17, 536 ± 61 min) or MII-SCC+MI oocytes (86%, 37/43, 563 ± 94 min). Therefore, the fusion procedure had little adverse effect on meiotic progression (Figures 1B and S1A).

In 61% (26/43) of cases, MII-SCCs formed a single spindle together with the host MI-SCCs. In 92% of these (24/26), dyads transferred to MI oocytes disjoined to form individual chromatids simultaneous with the conversion of bivalents into dyads (Figures 1C and 1E; Video S1). Two oocytes failed to undergo chromosome segregation and were arrested at MI. In those oocytes in which the MII-SCC remained separate from the host MI-SCC (39%, 17/43), dyads disjoined to form individual chromatids, and this process coincided with conversion of bivalents to dyads on the host MI spindle (Figures 1D and 1E; Video S1). This simultaneous double-chromosome segregation took place in 76% (13/17) of these oocytes. Most of these double divisions were accompanied by cytokinesis, producing two polar bodies. However, in 2 out of 17 (12%) only one of the two SCCs triggered cytokinesis. Two conclusions can be drawn from these findings. First, despite presence of an MI cytoplasm conducive to co-orientation of maternal and paternal KTs associated with bivalents, dyad KTs bi-orient just as they do in MII oocytes. Therefore, an MI cytoplasm cannot induce co-orientation of sister KTs in dyads. Second, the cytoplasm of MI oocytes cannot protect from Separase the periCEN cohesin holding dyads together. Both the propensity to bi-orient and the susceptibility of periCEN cohesin to Separase are therefore properties associated with SCCs and not conferred by the cytoplasmic state.

Video S1. Dyads transferred to MI oocytes formed chromatids simultaneously with the conversion of bivalents into dyads, related to Figure 1 Time lapse confocal imaging of chromosome segregation in MII-SCC+MI, whose MII-SCC was fused (left) or separated (right) with a host MI spindle. H2B-mCHERRY (magenta), chromosomes. eGFP-CENPC (green), kinetochores (KTs). Movie corresponds to still images in Figures 1C and 1D.

A possible reason why bi-orientation persists after transferring dyads to MI oocytes is that connections between KTs and microtubules established in MII persist throughout transfer process and are subsequently maintained until anaphase is initiated. To exclude this possibility, we transferred MII SCCs into GV oocytes (MII-SCC+GV), a procedure that leads to dissolution of pre-existing microtubule-KT attachments (Figures S1B–S1D; Video S2). Under these conditions, transferred dyads and host bivalents invariably (20/20) aligned on a single spindle, but anaphase was initiated in only 25% of oocytes (5/20) compared with 55% (6/11) when MII cytoplasts were fused to GV oocytes (MIIcyt+GV). The failure of MII-SCC+GV oocytes to undergo anaphase was only observed when they were imaged microscopically (Figure S1E), an effect exacerbated by presence of additional chromosomes in GV oocytes. Importantly, even in oocytes that failed to undergo anaphase, the vast majority of dyads that had been associated with spindles are bi-oriented (Figure S1F; Video S2). Crucially, in the five oocytes that underwent anaphase, their dyads disjoined to form individual chromatids (Figure S1D), confirming that their bi-orientation was functional. Moreover, both MII-SCC+GV and MIIcyt+GV oocytes underwent the second meiotic division as efficiently as non-transferred oocytes (Figure S1G), further suggesting that transferred MII cytoplasm does not affect the behavior of chromosomes. Thus, the KTs of dyads fail to co-orient even if all KT attachments are made anew when GV oocytes enter meiosis I.

Video S2. Dyads transferred to GV oocytes formed chromatids after MI-MII transition, related to Figure 1 Time lapse confocal imaging of chromosome segregation in MII-SCC+GV. Sister kinetochores in dyads failed to co-orient even though all kinetochore (KT) attachments were made anew when GV oocytes entered meiosis I. H2B-mCHERRY (magenta), chromosomes. eGFP-CENPC (green), KTs. Movie corresponds to still images in Figures S1D and S1F.

### Bivalents retain their character when transferred to MII oocytes

**Figure 2:**
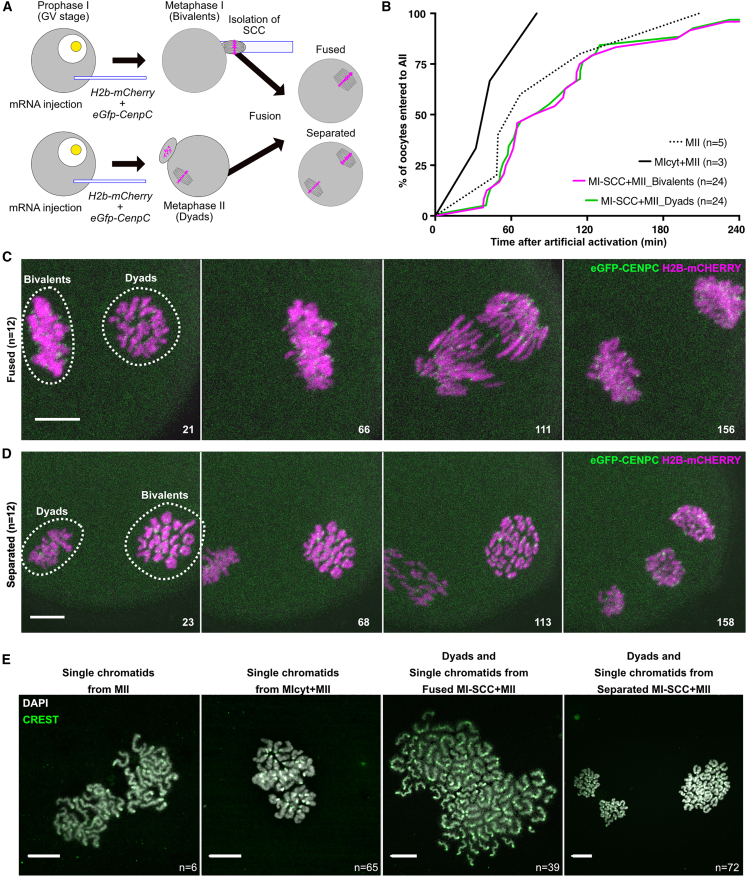
Determination of protection of cohesion or sister KT co-orientation is granted by bivalents rather than the factors from cytoplasm (A) Schematic of experiments showing an SCC containing bivalents is fused with an oocyte at MII (MI-SCC+MII). (B) Timing of bivalent segregation in MI-SCC+MII after artificial activation. (C and D) Live cell imaging of chromosome segregation in an MI-SCC+MII, whose MI-SCC was fused (C) or not fused with a host MII spindle (D) as in Figure 1C. Numbers indicate the time after artificial activation (min). Bars, 10 μm. (E) Dyad formation from transferred bivalents at AII (CREST: green; DAPI: gray). Bars, 15 μm. n, the numbers of oocytes analyzed.

Next, we addressed whether KT co-orientation and periCEN cohesin protection associated with bivalents is retained when they are transferred to MII oocytes (MI-SCC+MII) that are subsequently induced to undergo meiosis II by adding strontium (Figure 2A). The effect of MI cytoplasm transfer was assessed by preparing MII oocytes that had been fused with an MI cytoplast having a similar volume as an MI-SCC (MIcyt+MII). Chromosome segregation occurred in 24/39 (62%) MII oocytes fused with MI-SCC (MI-SCC+MII), a rate comparable with unfused MII oocytes (63%, 5/8) or MIcyt+MII oocytes (75%, 3/4). There was a modest delay in MI-SCC+MII oocytes (Figures 2B, S2A, and S2B). Because oocyte activation is triggered by a rapid influx of intracellular Ca^2+^ concentration released from the endoplasmic reticulum in the cytoplasm (Wakai and Fissore, 2013), the delay could be caused by a lower ratio of cytoplasm to chromosomes in MI-SCC+MII oocytes. In 13/39 (33%) MI-SCC+MII oocytes, the introduced SCC inter-mingled closely with the host SCC, forming a single spindle. Under these circumstances, 12/13 (92%) underwent chromosome segregation, during which MI bivalents were converted to dyads, whereas MII dyads were converted to individual chromatids (Figures 2C and 2E; Video S3). In the rest (26/39), the MI-SCC remained separate from the host MII-SCC. Of these, chromosome segregation took place in 46% (12/26), with bivalents being converted to dyads simultaneously with the conversion of dyads to individual chromatids (Figure 2D; Video S3). Thus, both KT co-orientation and protection of periCEN cohesion persist when bivalents are exposed to an MII cytoplasm. These results provide further confirmation that co-orientation and periCEN cohesin protection are properties not of the cytoplasm but of the chromosome. Because of similar findings with grasshopper spermatocytes (Nicklas, 1977; Paliulis and Nicklas, 2004), the chromosomal determination of KT and periCEN chromatin behavior appears to be a conserved feature, at least between insects and mammals.

Video S3. Bivalents transferred to MII oocytes converted to dyads simultaneously with the conversion of dyads to individual chromatids, related to Figure 2 Time lapse confocal imaging of chromosome segregation in MI-SCC+MII, whose MI-SCC was fused (left) or separated (right) with a host MII spindle. H2B-mCHERRY (magenta), chromosomes. eGFP-CENPC (green), KTs. Movie corresponds to still images in Figures 2C and 2D

### Separase cleavage at meiosis I is necessary for loss of periCEN cohesin protection and KT co-orientation

**Figure 3:**
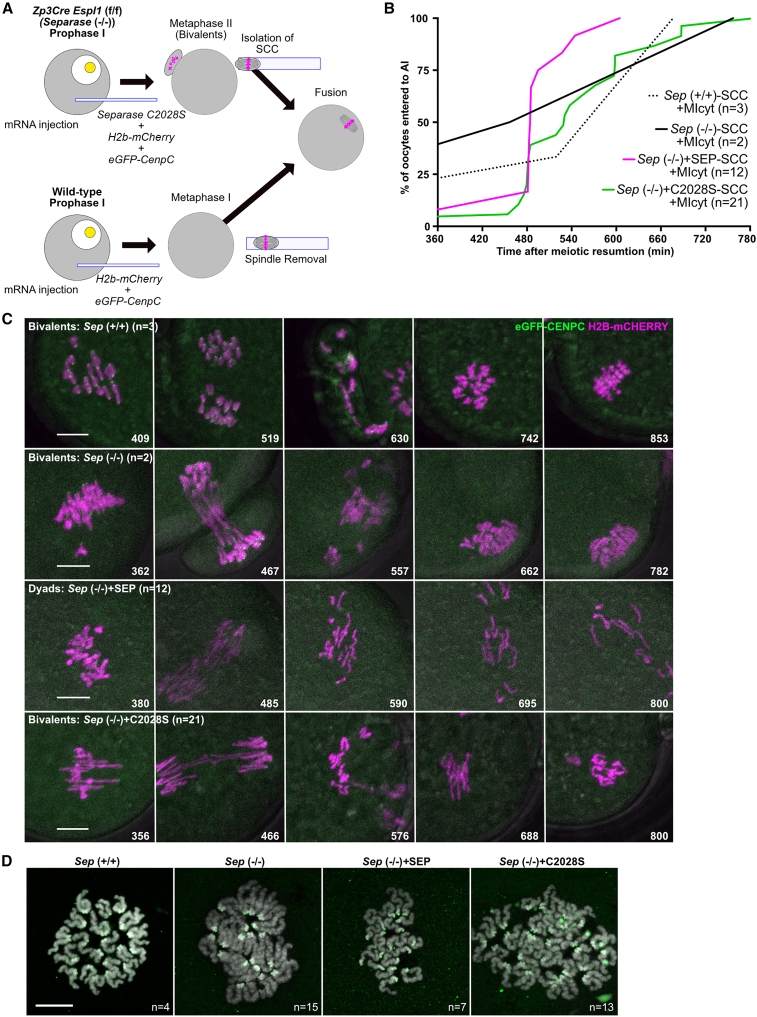
Cleavage of cohesin by separase is required for changing the status of bivalents for sister KT orientation and for a protection/deprotection of PeriCEN cohesion (A) Schematic of experiments showing that an SCC containing bivalents that have experienced all pathways during MI-MII transition, except REC8 cleavage by separase, is fused with an MI cytoplast. (B) Timing of bivalent segregation in MI cytoplasts (MIcyt). We prepared three kinds of bivalents whose SCC were fused with MIcyt. *Sep*(+/+), *Separase* (f/f). *Sep* (−/−), *Zp3Cre Separase* (f/f), *Sep* (−/−) C2028S, *Sep*(−/−) oocytes induced only cytokinesis by expression of catalytic-dead version of Separase C2028S. (C) Live cell imaging showing segregation of transferred bivalents in MI cytoplasts as in Figure 1C. Bars, 10 μm. (D) Transferred bivalents from *Sep* (−/−) oocytes formed dyads after the first meiotic division (CREST: green; DAPI: gray). Bar, 15 μm. n, the numbers of oocytes analyzed.

The previous experiments suggest that a change in the state of chromosomes rather than that of cytoplasm is responsible for the change in the behavior of KTs and periCEN cohesin upon completion of meiosis I. REC8 cleavage by Separase is a well-known chromosomal process during MI-MII transition. To test whether this has a role, we used an oocyte-specific knockout mouse line *Zp3-Cre Espl1* (*Separase*) (f/f), hereafter referred to as *Sep* (−/−), and a *Separase* (f/f) mouse line as a control (*Sep* (+/+), Kudo et al. 2006). As previously reported, the lack of chiasmata resolution in oocytes from *Sep* (−/−) mice is accompanied by a failure to extrude the first polar body (in 43/44 or 98% of oocytes). Polar body extrusion was rescued in 57/105 (55%) oocytes by injecting Separase mRNA, a success rate comparable with wild-type controls (61%, 17/28). Injection of mRNAs encoding a catalytically inactive Separase (C2028S) also rescued polar body extrusion in 124/224 oocytes (55%) but did not restore chiasmata resolution (Figure S3A; Kudo et al. 2006). Thus, oocytes specifically defective in cleavage activity, but no other Separase functions, undergo most if not all cell-cycle events that normally accompany APC/C activation during meiosis I, producing cells that contain bivalents instead of dyads. To address whether Separase cleavage activity is required to convert a chromosome state from MI to MII, the SCC from a *Sep* (−/−) *C2028S* oocyte that had extruded a polar body at MI was transferred to a wild-type MI oocyte, whose bivalents had been removed (Figure 3A). Crucially, bivalents on this SCC were converted to dyads with kinetics similar to those of *Sep* (+/+) oocytes (Figures 3B–3D; Video S4). Moreover, the dyads created were subsequently converted to individual chromatids when a second meiotic division was triggered through artificial activation (Figures S3B–S3D; Video S5). This result implies that cleavage of an SCC-associated protein by Separase is necessary to destroy co-orientation and protection of periCEN cohesion.

Video S4. Cleavage of an SCC-associated protein by separase is necessary to destroy co-orientation and protection of periCEN cohesion, related to Figure 3 Time lapse confocal imaging of bivalent segregation in MIcyt. We prepared three kinds of bivalents whose SCC were fused with MIcyt. *Sep (+/+), Separase (f/f)*. *Sep* (−/−), *Zp3Cre Separase* (f/f), *Sep (*−*/*−*)* C2028S, *Sep(*−*/*−*)* oocytes induced only cytokinesis by expression of catalytic-dead version of Separase C2028S. H2B-mCHERRY (magenta), chromosomes. eGFP-CENPC (green), KTs. Movie corresponds to still images in Figure 3C

Video S5. Dyads that have undergone two successive meiotic divisions are converted to single chromatids after artificial activation, related to Figure 3 Time lapse confocal imaging of dyads that have successfully undergone three successive divisions after artificial activation. *Sep* (−/−)+SEP: *Sep* (−/−)+SEP-SCC+MIcyt, *Sep* (−/−)+C2028S: *Sep* (−/−)+C2028S-SCC+MIcyt. H2B-mCHERRY (magenta), chromosomes. eGFP-CENPC (green), KTs. Movie corresponds to still images in Figure S3C.

### Different kinetics of REC8 cleavage along chromosome arms, at centromeres, and within periCEN chromatin are conferred by SGOL2

**Figure 4:**
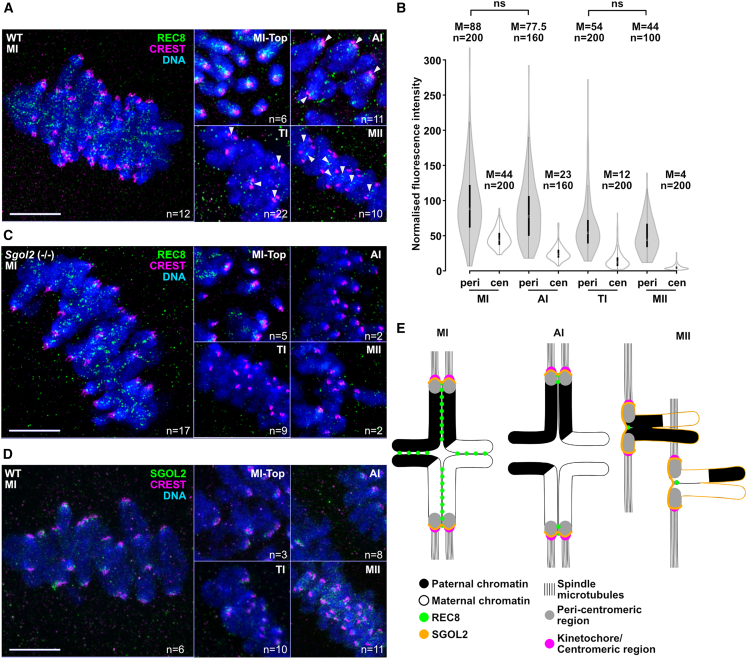
SGOL2 determines the dynamics of REC8 cleavage of arms, centromeres, and peri-centromeres during the first meiotic division (A) Representative super-resolution 3D-SIM images showing REC8 localization in wild type (WT) oocytes (REC8, green; CREST, magenta; DAPI, blue). The population of REC8 localized between sister KTs was observed from the top of spindle (MI-top). Arrowheads, REC8 at TI and MII. Bar, 5 μm. (B) Quantification of REC8 fluorescence intensity at periCEN (peri) and CEN (cen) regions during MI-MII transition. Only non-significance (ns) was shown. M, median. (C and D) REC8 localization in *Sgol2* (−/−) oocytes (C) and SGOL2 localization in WT oocytes (D) (REC8 or SGOL2, green; CREST, magenta; DAPI, blue). Bars, 5 μm. (E) Schematic of results showing REC8 (green) and SGOL2 (orange) localization during MI-MII transition. n, the numbers of oocytes/regions analyzed.

The preceding experiment raises the possibility that REC8 cleavage by Separase during meiosis I destroys periCEN cohesin protection, as well as KT co-orientation. To explore whether CEN Rec8 is its key target, we used super-resolution 3D structured illumination microscopy (3D-SIM) to observe in greater detail location of REC8-containing cohesin as oocytes undergo the first meiotic division (Lee et al., 2003). We located centromeres using CREST (calcinosis, Raynaud’s phenomenon, esophageal dysmotility, sclerodactyly, and telangiectasia) autoimmune serum and periCEN chromatin using antibodies against histone H3 tri-methylated at lysine 9 (H3K9me3) and topoisomerase II (Figure S4, Broccoli et al. (1990); Lee et al. (2011); Probst et al. 2007). In mice, centromeres are localized at the distal tips of each bivalent (Figures 4A and S4A). PeriCEN chromatin organized around axes associated with each sister chromatid is located just proximal to centromeres, with an axial length of 1.11 μm (Figures S4A and S4B). During MI, REC8 was detected between sister chromatids throughout bivalents, including periCEN and CEN regions. Despite their co-orientation, sister KTs are bisected by a zone of inter-chromatid REC8 when observed at the improved resolution made possible by 3D-SIM. This is particularly clear when the bivalent is observed from the spindle pole to which it is attached (Figure 4A MI-top). In other words, it is possible to detect a specific population of REC8 that is in an appropriate position to hold sister KTs together. Soon after the onset of anaphase I (AI), REC8 disappears from chromosome arms, which leads to loss of sister chromatid cohesion. This resolves the chiasmata and permits traction of dyads toward poles. Crucially, REC8 persists during AI not only throughout periCEN chromatin but also frequently within CEN regions (Figures 4A and 4B, AI). Some also persists at both locations until TI, during which a small amount remains sandwiched between closely apposed sister KTs (Figures 4A and 4B, TI). However, by the time cells enter MII, sister KTs are pulled several microns apart, and REC8, albeit in greatly reduced amounts, persists only in the periCEN regions that hold dyads together (Figures 4A and 4B, MII). These observations suggest that there are three populations of chromosomal REC8: arm, periCEN, and CEN. The arm population disappears at the onset of AI, whereas the CEN one only fully disappears after TI. In contrast, the periCEN population persists until the division of meiosis II, albeit only with reduced levels after TI. All three populations of REC8 persist on bivalents when *Sep* (−/−) *C2028S* oocytes attempt the first meiotic division (Figure S3A, MI) but disappear simultaneously at the onset of AI in *Sgol2 (*−*/*−*)* oocytes (Figure 4C). This suggests that SGOL2 protects CEN, as well as periCEN REC8 from Separase. However, in the case of CEN REC8, SGOL2 merely delays cleavage until after TI. Surprisingly, SGOL2 accumulates to maximal levels, not within periCEN chromatin, but just proximal to co-oriented KTs from MI to TI (Figure 4D) and only accumulates to higher levels within periCEN chromatin during meiosis II. The high levels of SGLO2 next to KTs may help to explain how it delays cleavage of CEN REC8. However, it is less clear why SGLO2 fails to protect CEN REC8 when cells enter meiosis II but manages to protect at least some periCEN REC8. The degree of protection cannot be explained merely by the pattern of SGOL2 accumulation, which is consistent with the observation in a previous report (El Yakoubi et al., 2017).

### CEN REC8 facilitates sister KT co-orientation

**Figure 5:**
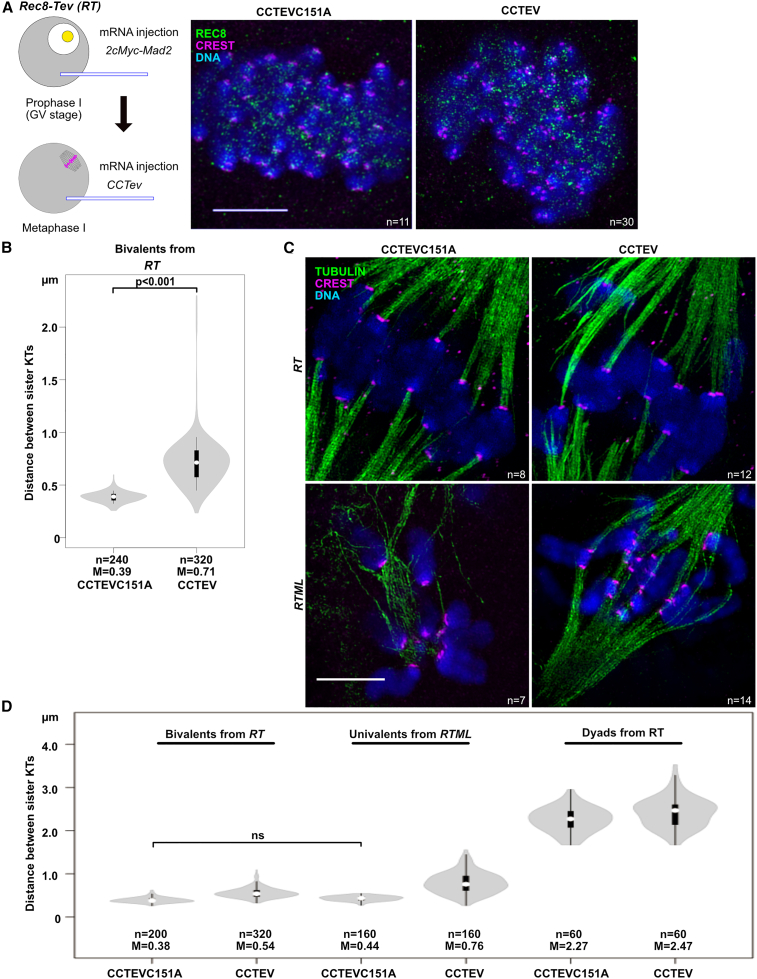
CEN cohesin is necessary for sister KT co-orientation of univalents at MI (A) Left, Schematic of experiments showing that induction of CEN-specific cleavage in bivalents having REC8-TEV (RT) using CCTEV. Right, specific cleavage of CEN REC8-TEV 6 h after induction of CCTEV expression (REC8, green; CREST, magenta; DAPI, blue). CCTEVC151A, a catalytic-dead version of CCTEV. Bar, 5 μm. (B and D) Distance between sister KTs in bivalents from *Rec8-Tev* (*RT*) or in univalents from *Rec8-Tev Mlh1* (–/–) (*RTML*) 4–6 h after cleavage of CEN REC8-TEV by CCTEV. White circle, median (M). Only non-significance (ns) was shown in (D). (C) Representative images showing that bi-orientation of sister KTs in univalents from *RTML* oocytes, which were lost CEN cohesin (tubulin, green; CREST, magenta; DAPI, blue). Bar, 5 μm. n, the numbers of oocytes/KTs analyzed.

Separase destroys both co-orientation and protection of periCEN cohesin and cleaves both chromosome arm and CEN REC8 by the time cells enter meiosis II. Given this, either arm or CEN REC8 cleavage could trigger loss of co-orientation and periCEN protection. To address the role of CEN REC8 cleavage, we injected oocytes whose *Rec8* contains Tobacco Etch Virus (TEV) protease cleavage sites (*Rec8-Tev, RT*) with mRNAs that encode a fusion of the Cenp-C DNA binding sequence to the N terminus of TEV protease (*CCTEV*, Tachibana-Konwalski et al. 2013). By targeting CCTEV to centromeres, we aimed to cleave REC8-TEV at centromeres but not elsewhere on chromosomes. The specificity of CCTEV was determined by checking the localization of REC8 on bivalents in *RT* oocytes 4–6 h after injecting *CCTEV* mRNA at a concentration of 1 ng/μl (Figures 5A and 5B). To prevent these oocytes from initiating AI, which would be accompanied by Separase-mediated cleavage, they were injected at the GV stage with mRNA for MAD2, which activates the spindle assembly checkpoint, SAC, and prevents activation of APC/C (Wassmann et al., 2003). To check whether the effects of CCTEV are due to its cleavage activity and not due to some adventitious side effect, we injected a set of control oocytes with mRNA for catalytically dead version, *CCTEVC151A* (Phan et al., 2002).

6 h after mRNA injection, CCTEV, but not CCTEVC151A, caused disappearance of REC8 from centromeres but not from chromosome arms, which was accompanied by a distinct moving apart of sister KTs (Figures 5A and 5B). Sister KT separation reached a median of 0.71 μm, nearly twice as far as the 0.39 μm in control CCTEVC151A oocytes. Interestingly, sister KT co-orientation persisted despite clear loss of cohesion between sister KTs (Figures 5A and 5B). There are two potential explanations for this. One possibility is that residual CEN cohesin that has evaded CCTEV is sufficient to support co-orientation. Alternatively, once co-orientation has been established, chiasmata ensure that tension stabilizes this state even when CEN cohesin has been fully removed by CCTEV. To address the latter, we repeated the experiment using *RT Mlh1* (−/−) (*RTML*) oocytes, which are defective in recombination and contain univalents instead of bivalents (Tachibana-Konwalski et al., 2010). In univalents, due to co-orientation of sister KTs and lack of chiasmata holding maternal and paternal KTs together, their KTs cannot establish stable connections to microtubules. They instead move from pole to pole as they are pulled first one way and then another.

Univalents from *RTML* oocytes expressed with CCTEVC151A behaved in a similar fashion, but those expressed with CCTEV underwent efficient bi-orientation and aligned stably on the spindle midplate (Figure 5C). These observations confirm that CCTEV is highly specific because cohesion persists along the inter-chromatid axes of univalents, thereby enabling stable bi-orientation. They also demonstrate that in the absence of chiasmata, cleavage of CEN REC8 is sufficient to destroy co-orientation. Thus, CEN REC8 has an important role in co-orientation, but it is not necessary when chiasmata connect maternal and paternal sister KT pairs.

Separation between sister KTs induced by cleavage of CEN REC8 by CCTEV was significantly greater in oocytes containing univalents than those containing bivalents (Figure 5D). This is not surprising as spindle forces pull apart the former but not the latter. More interestingly, we noticed that sister KTs separate less on bi-orienting univalents from *RTML* MI oocytes expressed with CCTEV than on bi-orienting dyads from *RT* MII oocytes expressed with either CCTEV or CCTEVC151A. This suggests that Separase removes more cohesin, either from CEN or more likely from periCEN chromatin, than does CCTEV, implying that protection of periCEN cohesin by SGOL2 is only partial, a scenario consistent with the lower levels of REC8 associated with the periCEN chromatin of dyads compared with bivalents (Figure 4A).

**Figure 6:**
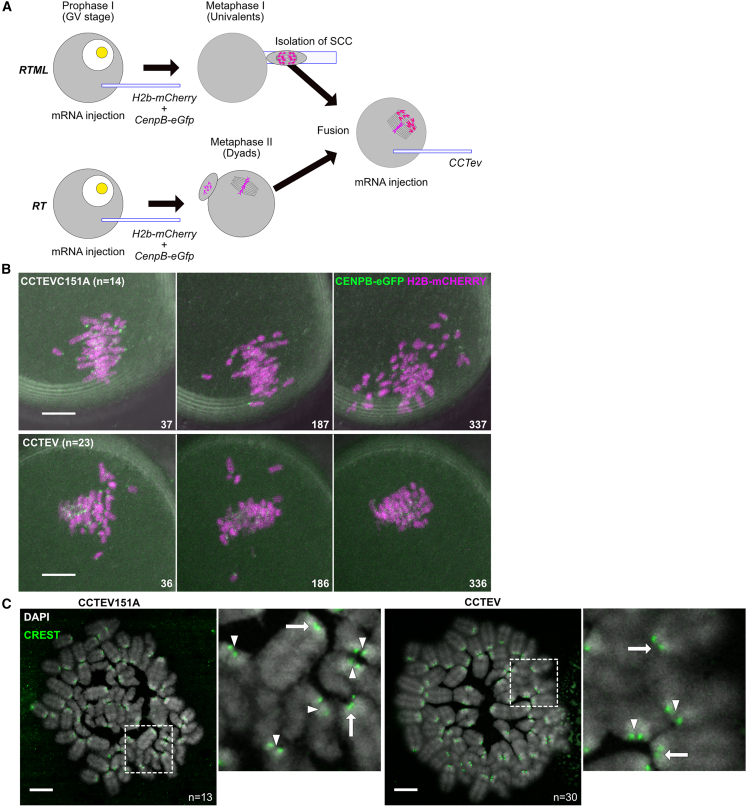
Specific cleavage of TEV-cleavable REC8 around centromeres by CCTEV (A and B) Schematic of experiments (A) and live cell imaging (B) showing the induction of sister KT bi-orientation in univalents by loss of CEN cohesin under the condition that periCEN cohesin in dyads is maintained. *RTML*, *Rec8-Tev Mlh1 (−/−)*. *RT*, *Rec8-Tev Mlh1(+/+)*. Numbers indicate the time after induction of CCTEV expression (min). H2B-mCherry (magenta), chromosomes. CENPB-eGFP (green), KTs. Bars, 10 μm. (C) Specific cleavage of CEN REC8-TEV by CCTEV (CREST: green; DAPI: gray). Images beside the whole chromosome spread show 4-fold magnification of the regions indicated in the dash-lined boxes. Univalents are attached to the chromosome arms (arrows) and the dyads are separated (arrowheads). Bars, 10 μm. n, the numbers of oocytes analyzed.

These experiments also revealed that separation between sister KTs of dyads was slightly but significantly greater when *RT* meiosis II oocytes were expressed with CCTEV than with CCTEVC151A (Figure 5D), raising the possibility that CCTEV may induce modest cleavage of periCEN, as well as CEN cohesin. Therefore, we set out to establish more precisely the specificity of CCTEV using a functional assay. To do this, we transferred an SCC containing univalents from an *RTML* MI oocyte to an MII *RT* oocyte containing dyads and analyzed the consequences of expression either CCTEV or CCTEVC151A (Figure 6A). If CCTEV cleaves CEN but not periCEN REC8, then CCTEV should induce bi-orientation of the transferred univalents but not induce disjunction of the host dyads. In the oocytes expressing CCTEVC151A, the transferred univalents moved back and forth between spindle poles, whereas the host dyads aligned at the spindle midzone, forming a metaphase plate. In contrast, in oocytes expressed with CCTEV, both sets of chromosomes bi-oriented at the spindle zone and remained there for several hours (Figure 6B; Video S6), implying that CCTEV destroys within the very same cell sufficient CEN REC8 to induce bi-orientation of univalents without adversely affecting the periCEN cohesion holding dyads together. Chromosome spreads from these oocytes confirmed that CCTEV’s cleavage activity caused a modest increase in the separation of sister KTs associated with the univalents but not to the extent observed with dyads (Figure 6C). In conclusion, by cleaving REC8, CCTEV triggers a change in the geometry of CEN DNA, which induces univalent sister KTs to bi-orient while leaving periCEN cohesion more or less intact.

Video S6. CCTEV cleaves CEN but not periCEN REC8, related to Figure 6 Time lapse confocal imaging showing that CCTEV destroys within the very same cell sufficient CEN REC8 to induce bi-orientation of univalents without adversely affecting the periCEN cohesion holding dyads together. H2B-mCHERRY (magenta), chromosomes. CENPB-eGFP (green), KTs. CCTEVC151A, a catalytic dead version of CCTEV. Movie corresponds to still images in Figure 6B

### CEN REC8 is necessary for efficient protection of periCEN REC8

**Figure 7:**
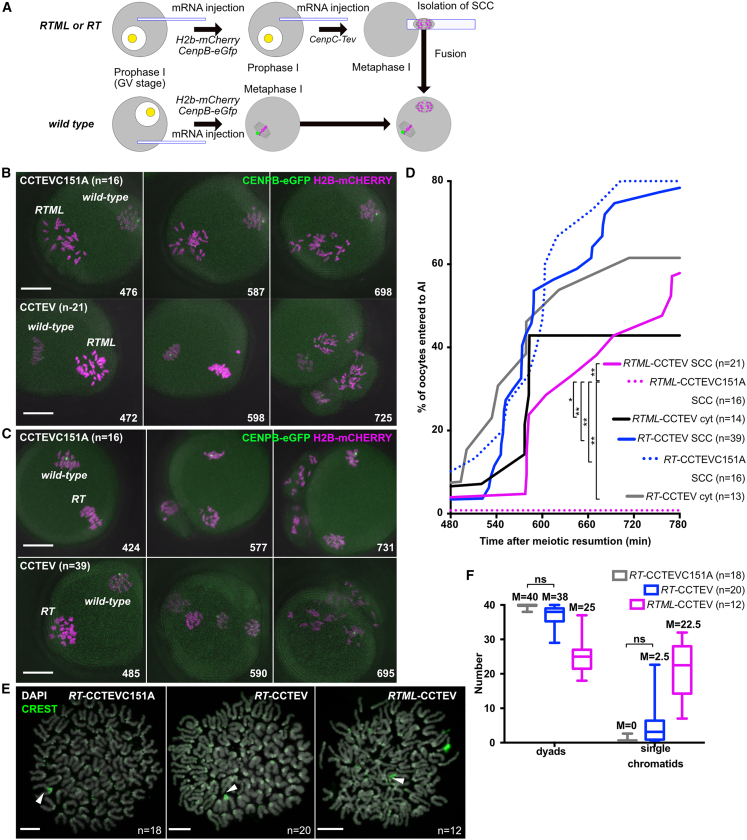
CEN cohesin is necessary for protecting PeriCEN cohesin at the first meiotic division (A) Schematic of experiments that demonstrate requirement of CEN cohesin for protection of periCEN cohesin. An MI oocyte (wild-type) from B6D2F1 mouse of which paternal chromosome 1 has two amplified CEN regions separated by a periCEN region thereby marked as a big bright signal by CENPB-eGFP. (B and C) Live cell imaging showing that univalents (*RTML*, B) or bivalents (*RT*, C) lacking CEN cohesin converted into single chromatids after meiosis I. Numbers indicate the time after meiotic resumption of wild-type oocytes (min). H2B-mCherry (magenta), chromosomes. CENPB-eGFP (green). Bars, 20 μm. (D) Segregation timing of bivalents (*RT*)/univalents (*RTML*) lacking CEN cohesin in an MI oocyte. Each group indicates wild-type oocytes fused with SCC (SCC) or cytoplasm (cyt) from *RT*/*RTML* after CEN cohesin cleavage by CCTEV. ^∗^p = 0.004, ^∗∗^p < 0.001. (E) Single chromatids formed after meiosis I from univalents without CEN cohesin (CREST, green; DAPI, gray). To confirm dyad formation of a paternal chromosome 1 (arrow heads) from a wild-type oocyte, cytokinesis was inhibited by cytochalasin B. Bars, 10 μm. (F) Number of dyad or single chromatid formation after meiosis I in a wild-type MI oocyte fused with an SCC from an *RT/RTML* oocyte previously expressed with CCTEV/CCTEVC151A. Only non-significance (ns) was shown. M, median. n, the numbers of oocytes analyzed.

Because Separase-mediated cleavage is required to destroy the protection of periCEN cohesin by SGOL2 and co-orientation, we next addressed whether CEN REC8 is necessary for protection, as well as co-orientation. To do this, we transferred to a wild-type oocyte an SCC containing univalents from *RTML* oocytes whose CEN REC8 had been cleaved by CCTEV (Figure 7A). To distinguish the transferred chromosomes from those of the host oocyte, the latter were isolated from B6D2F1 mice whose paternal chromosome 1 contains two CEN regions separated by a periCEN region (Mitchell et al., 1993), which associates with especially high levels of eGFP-CENPB. Fused oocytes containing univalents with CEN REC8, namely, those from *RTML* oocytes expressed with CCTEVC151A, failed to undergo the first meiotic division due to SAC activation (0/16, Figures 7B and 7D) (Tachibana-Konwalski et al., 2013). In contrast, those lacking CEN REC8, namely, those from *RTML* oocytes expressed with CCTEV, did so with an average timing of 664 ± 88 min (13/21, Figures 7B and 7D; Video S7) because bi-orientation of the univalents satisfied the SAC. Crucially, 75% of univalents were converted to individual chromatids at this division (Figures 7E and 7F, *RTML*-CCTEV). Indeed, had this not occurred, the division would not have been possible. Thus, removing CEN REC8 abolishes not only KT co-orientation but also the protection of periCEN REC8.

Video S7. Removing CEN REC8 abolishes not only KT co-orientation but also the protection of periCEN REC8, related to Figure 7 Time lapse confocal imaging showing that univalents (*RTML*) or bivalents (*RT*) lacking CEN cohesin converted into single chromatids after meiosis I. *RTML, Rec8-Tev Mlh1 (*−*/*−*). RT, Rec8-Tev Mlh1(+/+).* H2B-mCHERRY (magenta), chromosomes. CENPB-eGFP (green), KTs. Movie corresponds to still images in Figures 7B and 7C.

To address whether loss of protection is due to the bi-orientation of sister KTs caused by loss of CEN REC8, we repeated the experiment, this time transferring bivalents from *RT* oocytes previously expressing either CCTEVC151A or CCTEV. In the case of the former, all bivalents were converted to dyads when the oocytes underwent MI with an average timing of 586 ± 64 min (12/16, Figures 7C and 7D). However, 10% of bivalents whose CEN REC8 had been removed by CCTEV were converted to individual chromatids, not dyads, when the oocytes underwent the first meiotic division with an average timing of 609 ± 84 min (31/39, Figures 7C, 7D and 7F; Video S7). Since none of the bivalents underwent bi-orientation in this experiment, it would appear that CEN REC8 may have a direct role in conferring protection of periCEN REC8. Interestingly, this premature loss of periCEN cohesion was not accompanied by any change in the localization of SGOL2 and Meikin at MI (Figure S5).

To determine whether loss of sister KT co-orientation alone also contributes to deprotection of periCEN REC8, we analyzed the behavior of *Mlh1* (−/−) oocytes with intact REC8. Although most of their sister KTs co-orient, we noticed that one or two pairs of sister KTs bi-orient (Figure S6A). To test whether such chromosomes produce single chromatids when cells undergo the first meiotic division, we overcame the SAC-induced meiotic arrest caused by co-orientation of the majority of univalents by expressing a dominant-negative version of the APC/C activator CDC20, CDC20R132A whose inhibition of Mad2 binding to CDC20 creates a dominant active form (Figure S6C, Tachibana-Konwalski et al. 2013). CDC20 was used as an injection control, and the onset of AI was measured by monitoring degradation of SECURIN-eGFP (McGuinness et al., 2009). As expected, most univalents from *Mlh1* (−/−) oocytes injected with *Cdc20R132A* mRNA were converted into dyads during a division whose average timing was 412 ± 130 min (17/19). However, one or two univalents in each oocyte were instead converted to individual chromatids (Figures S6D and S6E; Video S8), implying precocious loss of periCEN cohesion. We presume that the single chromatids after division were originated from bi-oriented univalents. Therefore, it is possible that bi-orientation per se could also contribute to precocious loss of periCEN cohesion, but due to difficulties in tracking individual chromosomes throughout the division, we were unable to establish this with any certainty. Crucially, this effect was not due to the abnormal nature of the division induced by *Cdc20R132A*, because bivalents from *Mlh1 (+/+)* oocytes invariably produced dyads even when their first meiotic division was accelerated (Figures S6B and S6C; Video S8).

Video S8. Loss of sister KT co-orientation alone also contributes to deprotection of periCEN REC8, related to Figure 7 Time lapse confocal imaging showing the induction of first meiotic division by inhibition of the spindle check point using expression of CDC20R132A in *Mlh1*(−/−) oocytes. H2B-mCHERRY (magenta), chromosomes. SECURIN-eGFP (green), whose destruction indicates first anaphase entry and re-accumulation shows entry to meiosis II. Movie corresponds to still images in Figure S6C.

### SGOL2 is dispensable for maintaining periCEN cohesion during MII

How SGOL2 protects at least some periCEN REC8, but merely delays cleavage of CEN REC8 upon Separase activation at the onset of anaphase, remains an enigma since SGOL2 accumulates to much higher levels at centromeres during meiosis I than it does at peri-centromeres. It is nevertheless striking that SGOL2 does subsequently accumulate on periCEN sequences during MII. To address whether its presence at this location is necessary to maintain the periCEN cohesion holding dyads together prior to APC/C activation upon fertilization, we created a version of SGOL2 whose N terminus was fused to eGFP and that contains 3xTEV recognition sites at cysteine 706 (SGOL2-TEV706, Figure S7A). Expression of SGOL2-TEV706 at the GV stage had little effect on the frequency or timing of meiosis I of neither *Sgol2* (*+/+*) nor *Sgol2* (−*/*−) oocytes (Figures S7B and S7C) but fully restored the retention of periCEN cohesion in the latter (Figure S7E). Crucially, expression at MI of TEV protease abolished this ability (Figures S7B and S7D; Video S9), implying that cleavage around cysteine 706 inactivates SGLO2. In contrast, dyads created by expression of SGOL2-TEV706 in *Sgol2* (−*/*−) oocytes at the GV stage were unaffected when TEV were instead expressed during MII, implying that, although required during AI, SGOL2 is unnecessary for maintaining periCEN cohesion once oocytes enter MII (Figures S7D and S7E; Video S10), presumably because Separase is not active until fertilization reactivates the APC/C. Our observations are inconsistent with the suggestion that the dissipation of protection after meiosis I is mediated by the binding of a conserved histone chaperone SET/TAF-1b specifically at the periCEN region during meiosis II (Chambon et al., 2013; Wassmann, 2013).

Video S9. The TEV cleavage around cysteine 706 inactivates SGOL2, related to Figure 7 Time lapse confocal imaging showing that expression at MI of TEV protease abolished periCEN protection in *Sgol2 (*−*/*−*)* oocytes expressed with SGOL2-TEV706 from the GV stage. H2B-mCHERRY (magenta), chromosomes. eGFP-CENPC (green, left), KTs. SGOL2-TEV706 (green, middle & right), TEV cleavable SGOL2. TEVC151A, a catalytic dead version of TEV. Movie corresponds to still images in Figure S7B.

Video S10. SGOL2 is unnecessary for maintaining periCEN cohesion once oocytes enter MII, related to Figure 7 Time lapse confocal imaging showing the maintenance of dyads even after TEV cleavage of SGOL2-TEV706 (green) at MII. H2B-mCHERRY (magenta), chromosomes. TEVC151A, a catalytic dead version of TEV. Movie corresponds to still images in Figure S7D

## Discussion

An important advantage of the oocyte system is that it has been possible to address the molecular mechanism by which co-orientation and CEn cohesin protection are destroyed during the meiosis I/II transition using cytological and genetic manipulation. Our finding that cleavage activity mediated by Separase is essential implies that no other change brought about through activation of the APC/C or changes in the activity of protein kinases during the meiosis I/II transition is sufficient. Separase’s target could be REC8 (Kudo et al., 2006) and/or Meikin (Maier et al., 2021). Crucially, we show that cleavage of CEN REC8 is sufficient to destroy co-orientation, at least on univalent chromosomes. Thus, unlike Meikin, which is only required for efficient co-orientation (Kim et al., 2015), REC8 is essential and is therefore a better candidate. If Meikin cleavage were also needed, then it would have to be necessary for cleavage of CEN REC8, which has not been reported and does not seem plausible.

Loss of co-orientation and periCEN protection as cells enter meiosis II could be dependent on REC8 cleavage at any one of three chromosomal locations, namely, chromosome arms, periCEN, and CEN. We were able to test the effect of cleaving only CEN REC8, which was achieved by CCTEV expression in oocytes containing TEV-cleavable REC8. Despite increasing the separation between sister KTs, cleavage of CEN REC8 had little effect on the co-orientation of bivalents in MI. In contrast, it abolished co-orientation of sister KTs within univalents, causing them to bi-orient and subsequently disjoin into individual chromatids at AI. One explanation for this difference is that once established microtubule-KT attachments associated with bivalents are stable. In contrast, those associated with univalents are unstable, because co-orientation prevents the establishment of tension. This suggests that loss of co-orientation may require the transient detachment of KTs from microtubules. We note that co-orientation is also more readily lost in the absence of chiasmata in the fission yeast, *S*.*pombe*, (Hirose et al., 2011; Sakuno et al., 2009, 2011; Yokobayashi and Watanabe, 2005). Although it is entirely plausible that CEN cohesin co-orients KTs by holding together sister DNAs associated with KT proteins, our data do not exclude the possibility that another type of activity, such as loop extrusion altering the topology of CEN DNA (Davidson et al., 2019; Kim et al., 2019), might be involved.

### Limitations of the study

Our data also demonstrate that Separase-mediated cleavage, presumably of REC8, has a role in ablating protection by SGOL2 of periCEN cohesin, whose removal triggers AII. Two mysteries surround this phenomenon. The first concerns how protection is only conferred on some periCEN cohesion in a form that lasts till the onset of the second meiotic division. The protector, SGOL2, accumulates to far higher levels near centromeres than at periCEN sequences but merely delays cleavage of CEN REC8 until TI while protecting some periCEN REC8 beyond this point in time. It is possible that phosphorylation of CEN but not periCEN REC8 by a kinase recruited exclusively to centromeres overcomes de-phosphorylation mediated by PP2A. A good candidate is PLK1, which is recruited to KTs by Meikin binding to Cenp-C (Maier et al., 2021). If so, Meikin may have two opposing roles, promoting centromere co-orientation before Separase activation while subsequently helping Separase to destroy it. Both effects could be mediated directly through phosphorylation of REC8.

The second mystery concerns how protection within periCEN does eventually dissipate by the time Separase is reactivated at the onset of AII. Cleavage of CEN REC8 during meiosis I has a role in this dissipation as cleavage merely of CEN REC8 induces not only bi-orientation of univalents but also their transformation into individual chromatids at AI. However, it is also possible that limited cleavage of periCEN REC8 also has a role in dissipation. In other words, protection by SGOL2 may require a critical concentration of periCEN cohesin and partial cleavage of periCEN REC8 during meiosis I may lower its level below this critical level. Unfortunately, our experiments did not establish with any certainty whether the loss of periCEN protection caused by cleavage of CEN REC8 is a direct consequence of REC8 cleavage or an indirect consequence of the bi-orientation of sister KTs induced by this cleavage. What is clear is that bi-orientation per se does not remove SGLO2 from periCEN sequences, as has been proposed (Gómez et al., 2007; Lee et al., 2008), because SGOL2 accumulates at periCEN location to high levels, whereas sister KTs bi-orient during meiosis II.

## STAR★Methods

### Key resources table

**Table:** 

REAGENT or RESOURCE	SOURCE	IDENTIFIER
**Antibodies**		

Human polyclonal anti-Centromere	Antibodies Inc.	Cat#15-234; RRID: AB_2687472
Mouse polyclonal anti-REC8	Gift from Jibak Lee (Kobe U)(Lee et al., 2003)	N/A
Rabbit polyclonal anti-SGOL2	Gift from Yoshinori Watanabe (U Tokyo)(Lee et al., 2008)	N/A
Rabbit polyclonal anti-Meikin	Gift of Yoshinori Watanabe (U Tokyo) (Kim et al., 2015),	N/A
Rabbit monoclonal anti-Topoisomerase II (clone EPR5377)	Abcam	Cat#ab109524; RRID: AB_1085979
Rabbit polyclonal anti Histone H3 (tri methyl K9)	Abcam	Cat#ab8898; RRID: AB_306848
Mouse monoclonal anti-Tubulin (clone DM1A)	Sigma-Aldrich	Cat#T9026; RRID: AB_477593

**Chemicals, peptides, and recombinant proteins**		

M2	Sigma-Aldrich	Cat#M7167;
3-isobutyl-1-methylxanthine	Sigma-Aldrich	Cat#I7018; CAS#28822-58-4
Cytochalasin D	Sigma-Aldrich	Cat#C8273; CAS# 22144-77-0
Cytochalasin B	Sigma-Aldrich	Cat#C6762; CAS# 14930-96-2

**Critical commercial assays**		

GenomONE(TM)-CF EX SeV-E (HVJ-E) Cell Fusion Reagents	2B Scientific (Cosmo Bio)	Cat#ISK-CF-001-EX
mMESSAGEmMACHINE kit	Invitrogen (Ambion)	Cat#AM1340, AM1344&AM1348
RNeasy mini kit	Qiagen	Cat#74104

**Experimental models: Organisms/strains**		

Mouse: B6D2F1/Crl	Charles river	Strain code: 066
Mouse: C57BL/6JOlaHsd	Envigo	Order code: 057
Mouse: DBA/2OlaHsd	Envigo	Order code: 870
Mouse: *Zp3Cre Espl1(f/f)*: *Mixed background of C57BL/6J and 129/Sv*	Kudo et al. 2006	N/A
Mouse: *Rec8*-*Tev*: *Mixed background of 129P2Ola/Hsd*, *C57BL/6J and CBA/J*	Tachibana-Konwalski et al., 2010	N/A
Mouse: *Mlh1* (-/-) *Rec8*-*Tev*: *Mixed background of 129P2Ola/Hsd*, *C57BL/6J and CBA/J*	Tachibana-Konwalski et al., 2013	N/A

**Oligonucleotides**		

For insertion of 3xTEV recognition sites in SGOL2 Primer: *Bam*HI-*Sgol2*(1-706)-*Spe*I Forward: CGGGATCCGATGGAGTACCC AGGGATAAAAG	This paper	N/A
For insertion of 3xTEV recognition sites in SGOL2 Primer: *Bam*HI-*Sgol2*(1-706)-*Spe*I Reverse: GGACTAGTACATTTCACTCCA GAAATTACTTCTGTCTTG	This paper	N/A
For insertion of 3xTEV recognition sites in SGOL2 Primer: *Spe*I-*Sgol2*(707-1165)-*Xho*I Forward: GGACTAGTTTTAGTAATGA CCAAGGTGTTCATTGC	This paper	N/A
For insertion of 3xTEV recognition sites in SGOL2 Primer: *Spe*I-*Sgol2*(707-1165)-*Xho*I Reverse: CCGCTCGAGTTATCT CCTCATCTTGCTTCTAAGGC	This paper	N/A
Oligos for *Spe*I-3xTEV recognition sites-*Avr*II 5’actagtGAGAACCTCTATTTTCAA GGCCCGCGGGAGAATTTGTATTTCCA GGGTGGTAGCGAGAATTTGTATTT TCAGGGTcctagg3’	Tachibana-Konwalski et al., 2010	N/A

**Recombinant DNA**		

pRNA-*CenpB*-*eGFP*	Shelby et al., 1996	N/A
pRNA-*CenpC*-*Tev*/*TevC151A*	This paper	N/A
pRNA-*Cdc20*/*Cdc20R132A*	Tachibana-Konwalski et al., 2013	N/A
pGEMHE-*eGFP*-*CenpC1*	EUROSCARF	#P30659
pCMV-*eGfp*-*Sgol2*	Rattani et al., 2013	N/A
pRNA-*H2B*-*mCherry*	McGuinness et al., 2009	N/A
pRNA-*Securin*-*eGFP*	McGuinness et al., 2009	N/A
pRN3-*Espl1* (Separase)/*SeparaseC2028S*	Kudo et al., 2006	N/A
pCS2-*eGfp*-*Sgol2Tev706*	This paper	N/A

**Software and algorithms**		

Fiji (Image J)	Schindelin. et al., 2012	https://imagej.net/software/fiji/
Graphpad Prism	Graphpad Software	Prism 6 for Mac OS X
BoxPlotR		http://shiny.chemgrid.org/boxplotr/

### Resource availability

#### Lead contact

Further information and requests for resources and reagents should be directed to and will be fulfilled by the lead contact, Kim Nasmyth (ashley.nasmyth@bioch.ox.ac.uk).

#### Materials availability

There are restrictions to the availability of live mouse lines due to close down of the mouse project in Nasmyth lab, but frozen emrbyos are available. Plasmids generated in this study are available upon request from the lead contact.

### Experimental model and subject details

#### Mouse

All experimental procedures were approved by the University of Oxford local ethical review committee and licensed by the Home Office under the Animal (Scientific Procedures) Act 1986. Mice were housed in animal facilities at the University of Oxford, kept in individually ventilated cages and had free access to water and food with 12:12 hour light-dark cycle, controlled temperature (23-24°C), humidity and ventilation. For collection of wild-type oocytes, B6D2F1 female mice at 8-12 weeks were purchased from Charles River or crossed in house between the C57B6/J female and DBA2 male mouse strains from Envigo. *Zp3Cre Espl1(f/f)* (*Separase (-/-)*) female mice at 8-12 weeks were produced by crossing *Espl1(f/f)* females and *Zp3Cre* heterozygous males (Kudo et al., 2006). *Rec8-Tev* female mice at 8-12 weeks were maintained as homozygous (Tachibana-Konwalski et al., 2010). *Mlh1* (-/-) *Rec8-Tev* female mice at 7-10 weeks were obtained by crossing *Mlh1* (+/-) *Rec8-Tev* mice.

### Method details

#### Plasmid construction

The original plasmids for *CenpC-Tev/TevC151A*, pRNA-*CenpC-mCherry-Tev*/*TevC151A*, contain *mCherry* sequence between the sequences of *CenpC*-DNA binding domain and *Tev* (Tachibana-Konwalski et al., 2013). We improved the efficiency in kinetochore targeting of TEV by excising *mCherry* sequence and used the constructs, pRNA-*CenpC-Tev*/*TevC151A* in this study. For insertion of 3xTEV cleavage sites in SGOL2 at cysteine 706 (SGOL2-TEV706), cDNA fragment having SGOL2 residues 1-706 with flanking *Bam*HI and *Spe*I sites and that residues 707-1165 with flanking *Spe*I and *Xho*I sites were amplified from pCMV-*eGfp*-*Sgol2* (Rattani et al., 2013), then subcloned into pCS2*-eGfp* (Ogushi et al., 2017). After annealing and phosphorylation, oligos for 3x TEV cleavage sites with flanking *Spe*I and *Avr*II sites were inserted into pCS2-*eGfp*-*Sgol2* having a *Spe*I site just after cystein residue 706.

#### mRNA synthesis and microinjection

Capped mRNA constructs with a poly-A tail were transcribed using an mMESSAGEmMACHINE kit containing the appropriate RNA polymerase (Ambion). Following Turbo DNase I digestion for 15 min at 37°C, RNA was purified by using RNeasy mini kit (Qiagen), and resuspended in nuclease-free H_2_O. Each RNA was aliquoted and stored at −80°C until use. We used following plasmid cDNA encoding *Cdc20*, *Cdc20R132A*, *CenpB-eGfp* (Shelby et al., 1996), CenpC-Tev/-TevC151A, eGfp-CenpC, eGfp-Sgol2, eGfp-Sgol2-Tev706, H2B-mCherry, Securin-eGfp, Espl1 (Separase), SeparaseC2028S, Tev and TevC151A. In vitro-transcribed RNAs of 5 pl were microinjected under inverted microscope (Leica DM IRB) equipped with a micromanipulator (Narishige) and a pressure injector (WPI PV830 PicoPump) to the oocytes at following concentrations. *CenpB-eGfp* 100 ng/μl, *CenpC-Tev/-TevC151A* 1 ng/μl, *Tev/TevC151A 300 ng/μl*, *Cdc20/Cdc20R132A* 100 ng/μl, *eGfp-CenpC* 500 ng/μl, *eGfp-Sgol2/-Sgol2-Tev706* 300 ng/μl, *H2B-mCherry* 200 ng/μl, *Securin-eGfp* 500 ng/μl, *Separate/SeparaseC2028S* 100 ng/μl.

#### Spindle-chromosome complex (SCC) transfer

Oocytes at the germinal vesicle (GV)-stage were collected from ovaries of female mice at 8−12 weeks of age at 44−48 hours after injection with 7.5 IU equine chronic gonadotropin (eCG; Intervet). Fully grown GV-oocytes were released from ovarian follicles by puncture with needles in M2 (Sigma) supplemented with 200 μM 3-isobutyl-1-methylxanthine (IBMX; Sigma), and their cumulus cells were removed by gentle pipetting. GV-oocytes were cultured for 1 hour in M16 containing 200 μM IBMX (IBMX-M16) at 37°C under an atmosphere of 5% CO_2_, and then microinjected with mRNAs transcribed *in-vitro*. Injected oocytes were cultured for 1-2 hours in IBMX-M16 for expression of mRNAs, followed by the transfer into M16 for resumption of meiosis. SCC isolation was performed in M2 with 10-15 μg/ml cytochalasin D (Sigma) under an inverted microscope equipped with a micromanipulator and a PIEZO drive (Prime Tech PMAS-CT150) 4-6 hours (MI-SCC) or 12-16 hours (MII-SCC) after meiotic resumption. Firstly, an oocyte was positioned using a holding pipette so that the spindle was situated close to the 2 o’clock position. The zona pellucida next to a spindle was drilled with piezo pulses, an enucleation pipette was inserted through the opening hole, and a spindle surrounded by membrane was aspirated. Secondly, an enucleation pipette containing a spindle was move into the drop of HVJ-E extract (Ishihara Sangyo Kaisha) and HVJ-E extract with the double volume of spindle was aspirated. Finally, a spindle and HVJ-E extract were placed into the perivitelline space of the host oocyte. This construct was washed by M2 and transferred to IBMX-M16or M16, and incubated at 37°C in 5% CO_2_ for 30 min until fusion occurred. For further analysis, the fused oocytes of MII-SCC+MI were cultured in M16 and those of MII-SCC+GV were cultured for 2-4 hours in IBMX-M16 before they were released from their GV arrest and allowed to undergo meiosis.

#### Parthenogenetic activation

For parthenogenetic activation, oocytes 1 hour after fusion were artificially activated by treating them with activation medium, consisted of modified Tris-buffered medium (20 mM Tris, 113 mM NaCl, 3 mM KCl, 11 (2 glucose, 5 mM Na pyruvate, and 2 mg/ml BSA) with 10 mM SrCl_2_ for 4-6 hours. Because the movements associated with cytokinesis hinder chromosome imaging, cytokinesis was prevented by adding cytochalasin B (Sigma) at 5 μg/ml.

#### Live cell imaging

Following fusion or artificial activation, oocytes were transferred to 5-10 μl drops of M16 or activation medium on a glass-bottomed dish and placed in an incubator (Zeiss) at 37°C under 5% CO2 in air. Observation was performed under an inverted confocal microscope (Zeiss LSM780) equipped with a C-Apochromat 63×/1.2 water immersion objective with 3D multilocation tracking macro, which was kindly provided by J Ellenberg (EMBL, Germany, Rabut and Ellenberg 2004). We imaged 26 z-confocal sections every 2.0 μm of 512 × 512 pixel xy images at 15 min intervals.

#### Chromosome spread

The zona pellucida of oocytes or parthenotes was removed by treatment with 5 mg/ml protease in M2; then, the oocytes/parthenotes were placed on a glass slide that had been dipped in a solution of 1% PFA in distilled water, pH 9.2, containing 0.15% Triton X-100 and 3 mM dithiothreitol. Following overnight fixation in a humid chamber, the slide was dried for 30 min at room temperature. The samples were washed in PBS and then incubated with anti-centromere protein (Antibodies #15-234, 1:100) overnight at 4°C. After washing with PBS, the samples were incubated with Alexa-Fluor-488-conjugated goat anti-human IgG (Invitrogen) for 1.5 hours at room temperature and mounted with Vectashield containing DAPI (Vector Laboratories).

#### Whole-mount immunofluorescence

Basically, cultured oocytes were fixed in 2% paraformaldehyde (PFA) in PBS containing 0.1% Triton X-100 for 30 min. Only for microtubule staining, cultured oocytes were preincubated with ice-cold M2 for 10 min and proceeded to fixation as described above. After permeabilization with 0.1% Triton X-100 in PBS overnight at 4°C, oocytes were incubated with primary antibodies overnight at 4°C. Following three washes with PBS containing 0.1% polyvinyl alcohol (PVA), Alexa-Fluor-labelled secondary antibodies (Invitrogen) were used for the detection of signals and DNA was counterstained with 14.3 μM 4′,6-diamidino-2-phenylindole, dihydrochloride (DAPI, Invitrogen). The following primary antibodies were used: anti-REC8 (a kind gift from Jibak Lee, Kobe University, Japan; 1:100), anti-centromere protein (1:100), anti-SGOL2, anti-Meikin (kind gifts from Yoshinori Watanabe, University of Tokyo, Japan; 1:100 and 1:100, respectively), anti-TOPOII (Abcam, #ab109524, 1:100), anti-histone H3 tri-methylated at lysine 9 (H3K9me3; Abcam, #ab8898, 1:250-500) and anti-alpha Tubulin (Sigma, #T9026, 1:250-500). Samples were mounted on a slide with Vectashield (Vector Laboratories), covered with a No.1.5H (170 μm ± 5 μm) coverslip (Marienfield) and were imaged using a confocal laser scanning microscopy.

#### 3D structured illumination microscopy (3D-SIM)

3D-SIM was performed on a DeltaVision OMX V3 Blaze system (GE Healthcare) equipped with sCMOS cameras (PCO), and 405, 488 and 593nm lasers, using a 60x NA 1.3 silicone immersion objective lens (Olympus). To minimize artifacts due to spherical aberration when imaging entire oocytes of increased height (∼10-15 μm) and at extended depth (∼20-30 μm), the samples were mounted on a microscope slide with 63% Vectashield diluted in PBS to match the refractive index of the silicone immersion medium (RI=1.40), and then covered with a No.1.5H coverslip. The correction collar of the objective was before adjusted to obtain a symmetrical point spread function when imaging green beads at 488 nm excitation. Raw data was acquired with a z-distance of 125 nm and with 15 raw images per plane (5 phases, 3 angles). The raw data was reconstructed with SoftWoRx 6.2 (GE Healthcare) using channel-specifically measured optical transfer functions (OTFs) generated from ∼170nm diameter blue PS-Speck beads (ThermoFisher) and 100 nm diameter green and red FluoSphere beads (ThermoFisher), respectively, and Wiener filter setting 0.0040. Lateral color channel alignment was performed using a special image registration slide and algorithm provided by GE Healthcare. Correct 3D alignment was confirmed and refined in z by a custom test sample with two layers of 0.2 μm diameter TetraSpeck beads (ThermoFisher). The full-scale 32-bit reconstructed data was thresholded for each channel to the stack modal grey value (representing the center of the background intensity level) and converted to 16-bit composite tif-stacks using an in-house script in FIJI (Schindelin et al., 2012) before further processing. All 3D-SIM data was evaluated via SIMcheck, an open-source ImageJ plugin to assess SIM image quality.

### Quantification and statistical analysis

#### Quantification

For measuring sister kinetochore distance, we used 3D-SIM images of CREST signals which have been reconstructed and thresholded. Using plugins in FIJI, CREST signals were segmented by 3D segmentation and the sister kinetochore distance was measured by distance function in 3D manager. Axis lengths of pericentromeric regions were measured using images of H3K9me3 signals. Images were thresholded, and signals were segmented by 3D segmentation. Axial lengths were measured by 3D measure function (Fit Ellipse) in 3D manager. For quantification of REC8 signals, we mark the centromeric and pericentromeric regions based on CREST and intense DAPI signals and measure the intensity using quantification function (integrated density) in 3D manager. Intensity was normalised by max intensity of REC8 in each sample.

### Statistical analysis

For statistical analysis, we performed log-rank test (Figures 1B, 2B, 3B, and 7D), one-way ANOVA followed by Tukey's multiple comparison test (Figures 4B, 5D, and 7F) or Two-tailed t-test (Figure 5B). Unless otherwise stated in the figure legends, only when there is a significant difference between the data being compared is it indicated in the figure (*p*<0.01). All exact value of n from more than two independent experiments is included in the figures or figure legends.

#### Violin plots

Box limits indicate the 25th and 75th percentiles as determined by R software (BoxPlotR). Whiskers extend 1.5 times the interquartile range from the 25th and 75th percentiles. Polygons represent density estimates of data and extend to extreme values.

## Data Availability

All data reported in this paper will be shared by the lead contact upon request.

## Supplemental information

Document S1. Figures S1–S7

Document S2. Article plus supplemental information

## References

[bib1] Brar G.A., Kiburz B.M., Zhang Y., Kim J.-E., White F., Amon A. (2006). Rec8 phosphorylation and recombination promote the step-wise loss of cohesins in meiosis. Nature.

[bib2] Broccoli D., Miller O.J., Miller D.A. (1990). Relationship of mouse minor satellite DNA to centromere activity. Cytogenet. Cell Genet..

[bib3] Chambon J.-P., Touati S.A., Berneau S., Cladière D., Hebras C., Groeme R., McDougall A., Wassmann K. (2013). The PP2A inhibitor I2PP2A is essential for sister chromatid segregation in oocyte meiosis II. Curr. Biol..

[bib4] Corbett K.D., Harrison S.C. (2012). Molecular architecture of the yeast monopolin complex. Cell Rep..

[bib5] Davidson I.F., Bauer B., Goetz D., Tang W., Wutz G., Peters J.-M. (2019). DNA loop extrusion by human cohesin. Science.

[bib6] El Yakoubi W., Buffin E., Cladière D., Gryaznova Y., Berenguer I., Touati S.A., Gómez R., Suja J.A., van Deursen J.M., Wassmann K. (2017). Mps1 kinase-dependent Sgo2 centromere localisation mediates cohesin protection in mouse oocyte meiosis I. Nat. Commun..

[bib8] Gómez R., Valdeolmillos A., Parra M.T., Viera A., Carreiro C., Roncal F., Rufas J.S., Barbero J.L., Suja J.A. (2007). Mammalian SGO2 appears at the inner centromere domain and redistributes depending on tension across centromeres during meiosis II and mitosis. EMBO Rep..

[bib9] Gregan J., Rumpf C., Li Z., Cipak L. (2008). What makes centromeric cohesion resistant to separase cleavage during meiosis I but not during meiosis II?. Cell Cycle.

[bib10] Hirose Y., Suzuki R., Ohba T., Hinohara Y., Matsuhara H., Yoshida M., Itabashi Y., Murakami H., Yamamoto A. (2011). Chiasmata promote monopolar attachment of sister chromatids and their co-segregation toward the proper pole during meiosis I. PLoS Genet..

[bib11] Jonak K., Zagoriy I., Oz T., Graf P., Rojas J., Mengoli V., Zachariae W. (2017). APC/C-Cdc20 mediates deprotection of centromeric cohesin at meiosis II in yeast. Cell Cycle.

[bib12] Katis V.L., Lipp J.J., Imre R., Bogdanova A., Okaz E., Habermann B., Mechtler K., Nasmyth K., Zachariae W. (2010). Rec8 phosphorylation by casein kinase 1 and Cdc7-Dbf4 kinase regulates cohesin cleavage by separase during meiosis. Dev. Cell.

[bib13] Katis V.L., Matos J., Mori S., Shirahige K., Zachariae W., Nasmyth K. (2004). Spo13 facilitates monopolin recruitment to kinetochores and regulates maintenance of centromeric cohesion during yeast meiosis. Curr. Biol..

[bib14] Kim J., Ishiguro K.I., Nambu A., Akiyoshi B., Yokobayashi S., Kagami A., Ishiguro T., Pendas A.M., Takeda N., Sakakibara Y. (2015). Meikin is a conserved regulator of meiosis-I-specific kinetochore function. Nature.

[bib15] Kim Y., Shi Z., Zhang H., Finkelstein I.J., Yu H. (2019). Human cohesin compacts DNA by loop extrusion. Science.

[bib16] Kitajima T.S., Sakuno T., Ishiguro K.I., Iemura S.I., Natsume T., Kawashima S.A., Watanabe Y. (2006). Shugoshin collaborates with protein phosphatase 2A to protect cohesin. Nature.

[bib17] Klapholz S., Esposito R.E. (1980). Recombination and chromosome segregation during the single division meiosis in SPO12-1 and SPO13-1 diploids. Genetics.

[bib18] Kudo N.R., Wassmann K., Anger M., Schuh M., Wirth K.G., Xu H., Helmhart W., Kudo H., Mckay M., Maro B. (2006). Resolution of chiasmata in oocytes requires separase-mediated proteolysis. Cell.

[bib19] Lee B.H., Kiburz B.M., Amon A. (2004). Spo13 maintains centromeric cohesion and kinetochore coorientation during meiosis I. Curr. Biol..

[bib20] Lee J., Iwai T., Yokota T., Yamashita M. (2003). Temporally and spatially selective loss of Rec8 protein from meiotic chromosomes during mammalian meiosis. J. Cell Sci..

[bib21] Lee J., Kitajima T.S., Tanno Y., Yoshida K., Morita T., Miyano T., Miyake M., Watanabe Y. (2008). Unified mode of centromeric protection by shugoshin in mammalian oocytes and somatic cells. Nat. Cell Biol..

[bib22] Lee J., Ogushi S., Saitou M., Hirano T. (2011). Condensins I and II are essential for construction of bivalent chromosomes in mouse oocytes. Mol. Biol. Cell.

[bib23] Maier N.K., Ma J., Lampson M.A., Cheeseman I.M. (2021). Separase cleaves the kinetochore protein Meikin at the meiosis I/II transition. Dev. Cell.

[bib24] Marston A.L. (2015). Shugoshins: tension-sensitive pericentromeric adaptors safeguarding chromosome segregation. Mol. Cell. Biol..

[bib25] McGuinness B.E., Anger M., Kouznetsova A., Gil-Bernabé A.M., Helmhart W., Kudo N.R., Wuensche A., Taylor S., Hoog C., Novak B., Nasmyth K. (2009). Regulation of APC/C activity in oocytes by a Bub1-dependent spindle assembly checkpoint. Curr. Biol..

[bib26] McKinley K.L., Cheeseman I.M. (2016). The molecular basis for centromere identity and function. Nat. Rev. Mol. Cell Biol..

[bib28] Mitchell A.R., Nicol L., Malloy P., Kipling D. (1993). Novel structural organisation of a Mus musculus DBA/2 chromosome shows a fixed position for the centromere. J. Cell Sci..

[bib29] Nasmyth K. (2015). A meiotic mystery: how sister kinetochores avoid being pulled in opposite directions during the first division. BioEssays.

[bib30] Nicklas R.B. (1977). Chromosome distribution: experiments on cell hybrids and in vitro. Philos. Trans. R. Soc. Lond. B Biol. Sci..

[bib31] Ogushi S., Yamagata K., Obuse C., Furuta K., Wakayama T., Matzuk M.M., Saitou M. (2017). Reconstitution of the oocyte nucleolus in mice through a single nucleolar protein. NPM2. J. Cell Sci..

[bib32] Paliulis L.V., Nicklas R.B. (2004). Micromanipulation of chromosomes reveals that cohesion release during cell division is gradual and does not require tension. Curr. Biol..

[bib33] Phan J., Zdanov A., Evdokimov A.G., Tropea J.E., Peters H.K., Kapust R.B., Li M., Wlodawer A., Waugh D.S. (2002). Structural basis for the substrate specificity of tobacco etch virus protease. J. Biol. Chem..

[bib34] Plowman R., Singh N., Tromer E.C., Payan A., Duro E., Spanos C., Rappsilber J., Snel B., Kops G.J.P.L., Corbett K.D., Marston A.L. (2019). The molecular basis of monopolin recruitment to the kinetochore. Chromosoma.

[bib35] Probst A.V., Santos F., Reik W., Almouzni G., Dean W. (2007). Structural differences in centromeric heterochromatin are spatially reconciled on fertilisation in the mouse zygote. Chromosoma.

[bib36] Rabitsch K.P., Petronczki M., Javerzat J.P., Genier S., Chwalla B., Schleiffer A., Tanaka T.U., Nasmyth K. (2003). Kinetochore recruitment of two nucleolar proteins is required for homolog segregation in meiosis I. Dev. Cell.

[bib37] Rabut G., Ellenberg J. (2004). Automatic real-time three-dimensional cell tracking by fluorescence microscopy. J. Microsc..

[bib38] Rattani A., Wolna M., Ploquin M., Helmhart W., Morrone S., Mayer B., Godwin J., Xu W., Stemmann O., Pendas A., Nasmyth K. (2013). Sgol2 provides a regulatory platform that coordinates essential cell cycle processes during meiosis I in oocytes. Elife.

[bib39] Riedel C.G., Katis V.L., Katou Y., Mori S., Itoh T., Helmhart W., Gálová M., Petronczki M., Gregan J., Cetin B. (2006). Protein phosphatase 2A protects centromeric sister chromatid cohesion during meiosis I. Nature.

[bib40] Sakuno T., Tada K., Watanabe Y. (2009). Kinetochore geometry defined by cohesion within the centromere. Nature.

[bib41] Sakuno T., Tanaka K., Hauf S., Watanabe Y. (2011). Repositioning of aurora b promoted by chiasmata ensures sister chromatid mono-orientation in meiosis I. Dev. Cell.

[bib42] Schindelin J., Arganda-Carreras I., Frise E., Kaynig V., Longair M., Pietzsch T., Preibisch S., Rueden C., Saalfeld S., Schmid B. (2012). Fiji: an open-source platform for biological-image analysis. Nat. Methods.

[bib43] Shelby R.D., Hahn K.M., Sullivan K.F. (1996). Dynamic elastic behavior of alpha-satellite DNA domains visualized in situ in living human cells. J. Cell Biol..

[bib44] Shonn M.A., McCarroll R., Murray A.W. (2002). Spo13 protects meiotic cohesin at centromeres in meiosis I. Genes Dev.

[bib45] Tachibana-Konwalski K., Godwin J., Borsos M., Rattani A., Adams D.J., Nasmyth K. (2013). Spindle assembly checkpoint of oocytes depends on a kinetochore structure determined by cohesin in meiosis I. Curr. Biol..

[bib46] Tachibana-Konwalski K., Godwin J., Van Der Weyden L., Champion L., Kudo N.R., Adams D.J., Nasmyth K. (2010). Rec8-containing cohesin maintains bivalents without turnover during the growing phase of mouse oocytes. Genes Dev.

[bib47] Tóth A., Rabitsch K.P., Gálová M., Schleiffer A., Buonomo S.B.C., Nasmyth K. (2000). Functional genomics identifies monopolin: A kinetochore protein required for segregation of homologs during meiosis I. Cell.

[bib48] Touati S.A., Cladière D., Lister L.M., Leontiou I., Chambon J.-P., Rattani A., Böttger F., Stemmann O., Nasmyth K., Herbert M., Wassmann K. (2012). Cyclin A2 is required for sister chromatid segregation, but not separase control, in mouse oocyte meiosis. Cell Rep..

[bib49] Vissel B., Choo K.H. (1989). Mouse major (gamma) satellite DNA is highly conserved and organized into extremely long tandem arrays: implications for recombination between nonhomologous chromosomes. Genomics.

[bib50] Wakai T., Fissore R.A. (2013). Ca(2+) homeostasis and regulation of ER Ca(2+) in mammalian oocytes/eggs. Cell Calcium.

[bib51] Wang H.T., Frackman S., Kowalisyn J., Esposito R.E., Elder R. (1987). Developmental regulation of SPO13, a gene required for separation of homologous chromosomes at meiosis I. Mol. Cell. Biol..

[bib52] Wassmann K. (2013). Sister chromatid segregation in meiosis II : deprotection through phosphorylation. Cell Cycle.

[bib53] Wassmann K., Niault T., Maro B. (2003). Metaphase I arrest upon activation of the Mad2-dependent spindle checkpoint in mouse oocytes. Curr. Biol..

[bib54] Watanabe Y. (2012). Geometry and force behind kinetochore orientation: lessons from meiosis. Nat. Rev. Mol. Cell Biol..

[bib55] Watanabe Y., Nurse P. (1999). Cohesin Rec8 is required for reductional chromosome segregation at meiosis. Nature.

[bib56] Xu Z., Cetin B., Anger M., Cho U.S., Helmhart W., Nasmyth K., Xu W. (2009). Structure and function of the PP2A-shugoshin interaction. Mol. Cell.

[bib57] Yokobayashi S., Watanabe Y. (2005). The kinetochore protein Moa1 enables cohesion-mediated monopolar attachment at meiosis I. Cell.

