# Supplementary material for: Loss of sister kinetochore co-orientation and peri-centromeric cohesin protection after meiosis I depends on cleavage of centromeric REC8
Source: Dev Cell. 2021 Nov 22;56(22):3100–3114.e4. doi: 10.1016/j.devcel.2021.10.017 (PMC8629431; doi:10.1016/j.devcel.2021.10.017)
Supplement: Document S1. Figures S1–S7 [file mmc1.pdf]

Developmental Cell, Volume *56*

Supplemental information

Loss of sister kinetochore co-orientation and peri-centromeric cohesin protection after meiosis I depends on cleavage of centromeric REC8

Sugako Ogushi, Ahmed Rattani, Jonathan Godwin, Jean Metson, Lothar Schermelleh, and Kim Nasmyth

Figure S1

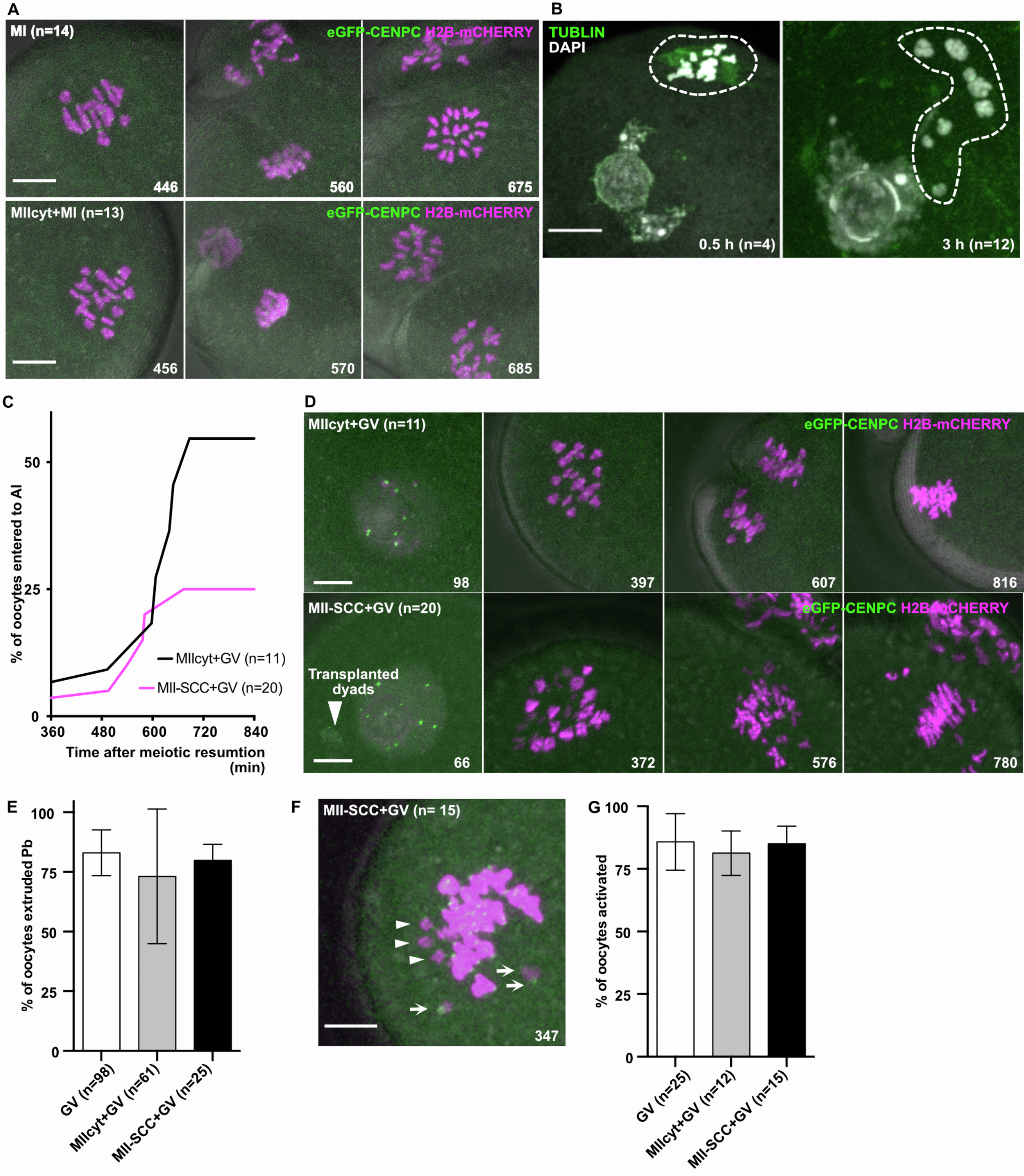
A
MI (n=14)
eGFP-CENPC H2B-mCHERRY
446
560
675
MIIcyt+MI (n=13)
eGFP-CENPC H2B-mCHERRY
456
570
685
B
TUBLIN
DAPI
0.5 h (n=4)
3 h (n=12)
C
% of oocytes entered to AI
Time after meiotic resumtion (min)
MIIcyt+GV (n=11)
MII-SCC+GV (n=20)
D
MIIcyt+GV (n=11)
eGFP-CENPC H2B-mCHERRY
98
397
607
816
MII-SCC+GV (n=20)
eGFP-CENPC H2B-mCHERRY
Transplanted dyads
66
372
576
780
E
% of oocytes extruded Pb
GV (n=98)
MIIcyt+GV (n=61)
MII-SCC+GV (n=25)
F
MII-SCC+GV (n= 15)
347
G
% of oocytes activated
GV (n=25)
MIIcyt+GV (n=12)
MII-SCC+GV (n=15)

Figure S2

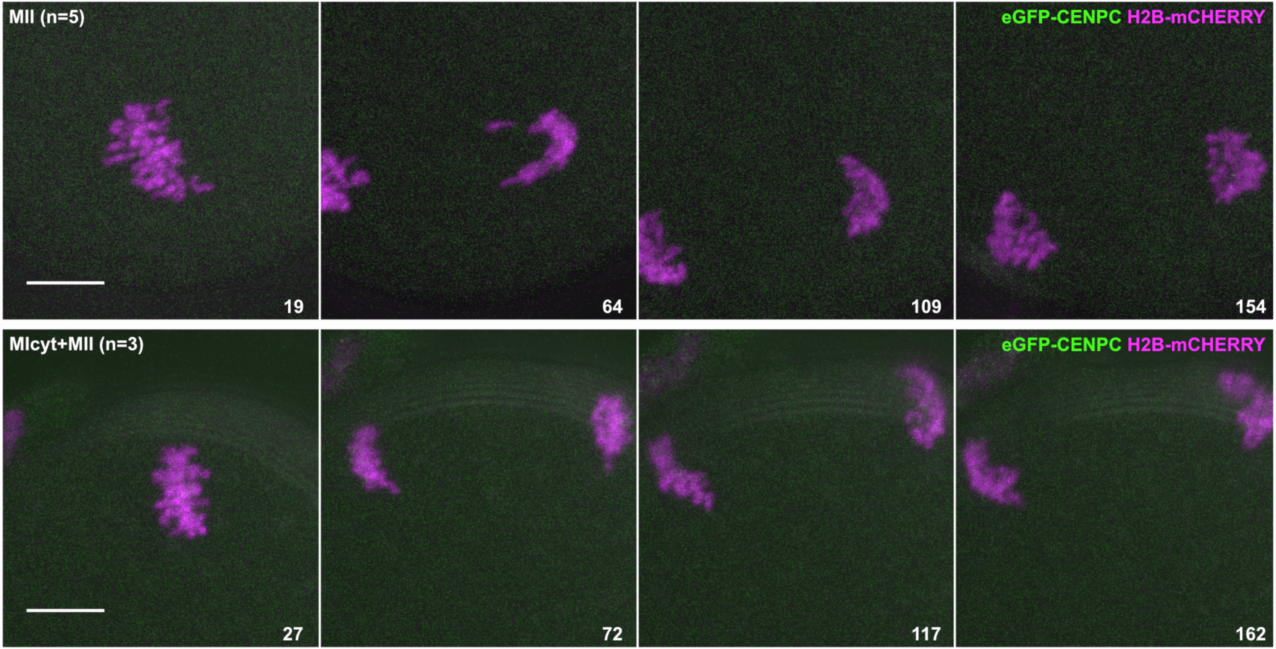
MII (n=5)
eGFP-CENPC H2B-mCHERRY
19
64
109
154
MIcyt+MII (n=3)
eGFP-CENPC H2B-mCHERRY
27
72
117
162

Figure S3

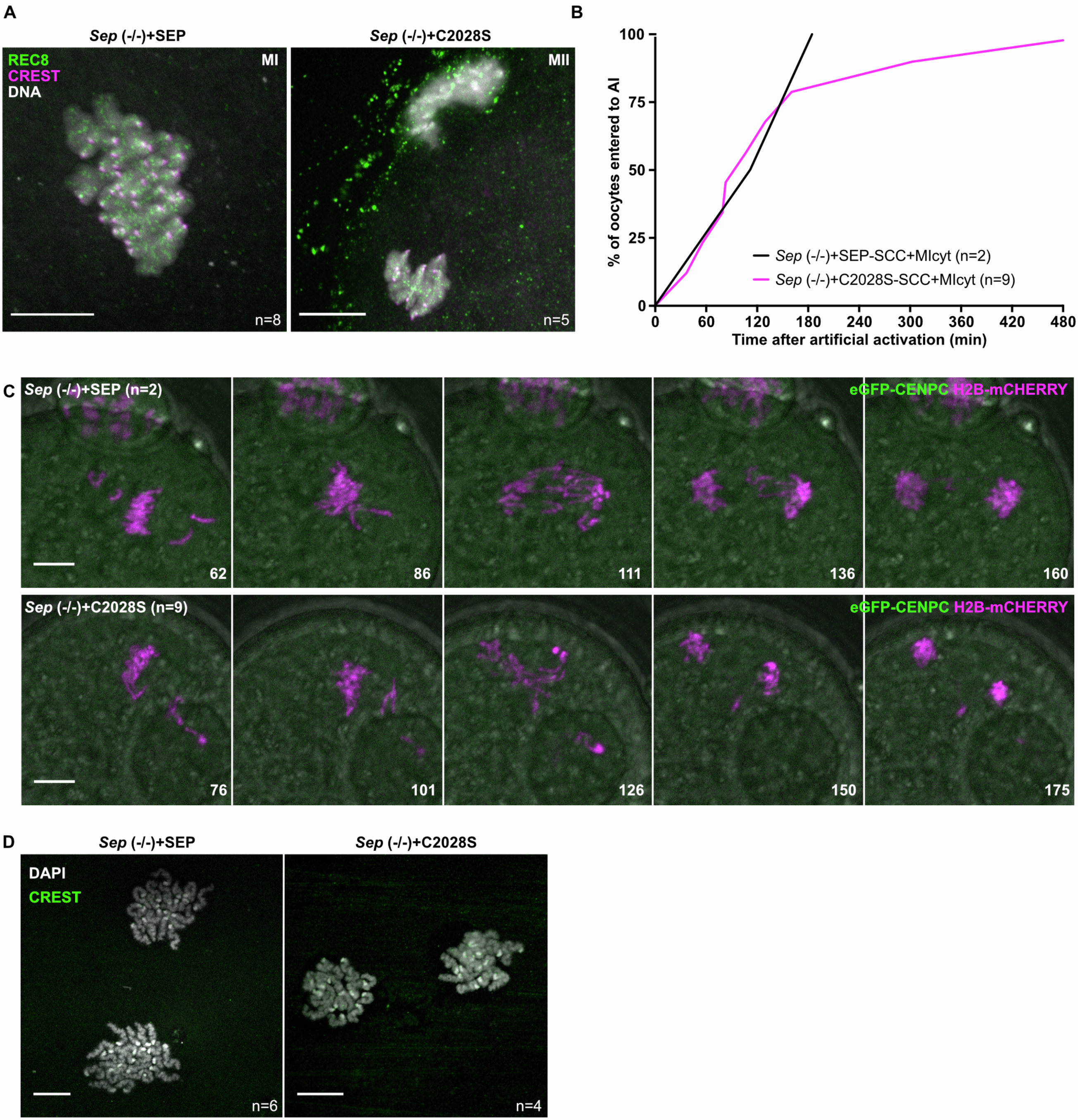
A
Sep (-/-)+SEP
Sep (-/-)+C2028S
REC8
CREST
DNA
MI
MII
n=8
n=5
B
% of oocytes entered to AI
Time after artificial activation (min)
Sep (-/-)+SEP-SCC+MIcyt (n=2)
Sep (-/-)+C2028S-SCC+MIcyt (n=9)
0
25
50
75
100
0
60
120
180
240
300
360
420
480
C
Sep (-/-)+SEP (n=2)
eGFP-CENPC H2B-mCHERRY
62
86
111
136
160
Sep (-/-)+C2028S (n=9)
eGFP-CENPC H2B-mCHERRY
76
101
126
150
175
D
Sep (-/-)+SEP
Sep (-/-)+C2028S
DAPI
CREST
n=6
n=4

Figure S4

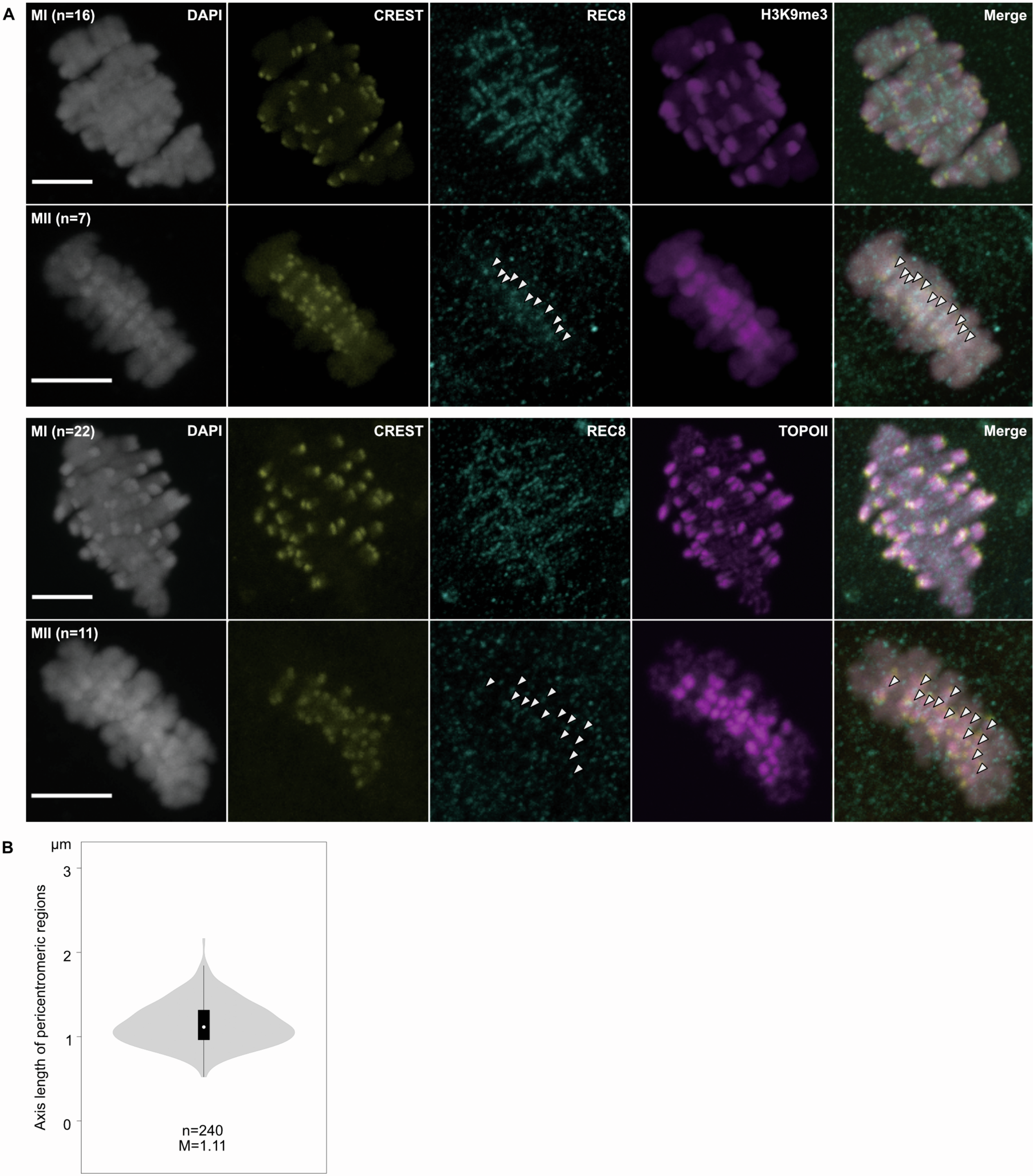
A
MI (n=16)
DAPI
CREST
REC8
H3K9me3
Merge
MII (n=7)
MI (n=22)
DAPI
CREST
REC8
TOPOII
Merge
MII (n=11)
B
µm
3
2
1
0
Axis length of pericentromeric regions
n=240
M=1.11

Figure S5

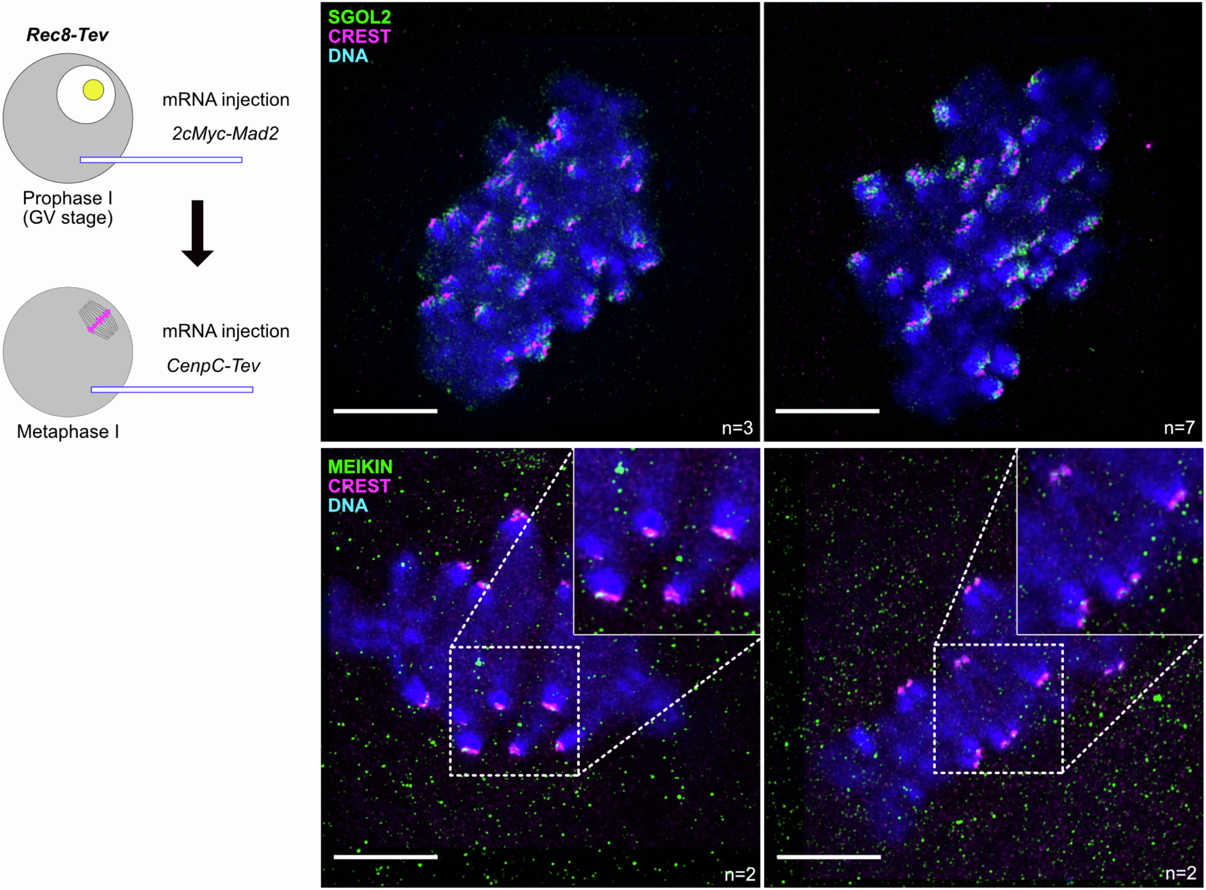

Rec8-Tev
mRNA injection
2cMyc-Mad2
Prophase I
(GV stage)
mRNA injection
CenpC-Tev
Metaphase I
CCTEVC151A
CCTEV
SGOL2
CREST
DNA
n=3
n=7
MEIKIN
CREST
DNA
n=2
n=2

Figure S6

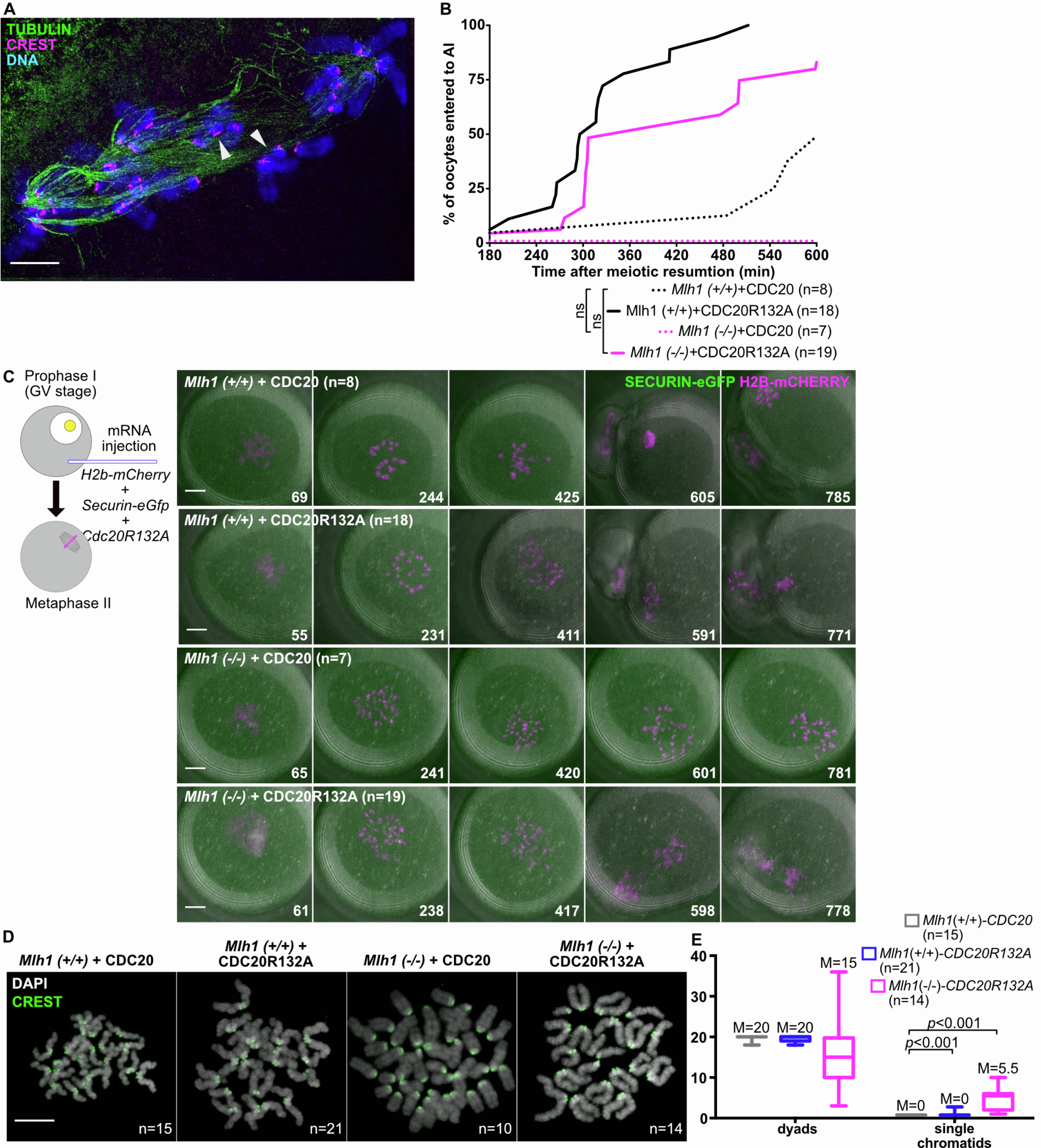

A
TUBULIN
CREST
DNA
B
% of oocytes entered to AI
100
75
50
25
0
180 240 300 360 420 480 540 600
Time after meiotic resumtion (min)
ns
ns
Mlh1 (+/+)+CDC20 (n=8)
Mlh1 (+/+)+CDC20R132A (n=18)
Mlh1 (-/-)+CDC20 (n=7)
Mlh1 (-/-)+CDC20R132A (n=19)
C
Prophase I
(GV stage)
mRNA injection
H2b-mCherry
+
Securin-eGfp
+
Cdc20R132A
Metaphase II
SECURIN-eGFP H2B-mCHERRY
Mlh1 (+/+) + CDC20 (n=8)
69 244 425 605 785
Mlh1 (+/+) + CDC20R132A (n=18)
55 231 411 591 771
Mlh1 (-/-) + CDC20 (n=7)
65 241 420 601 781
Mlh1 (-/-) + CDC20R132A (n=19)
61 238 417 598 778
D
Mlh1 (+/+) + CDC20
Mlh1 (+/+) + CDC20R132A
Mlh1 (-/-) + CDC20
Mlh1 (-/-) + CDC20R132A
DAPI
CREST
n=15
n=21
n=10
n=14
E
Mlh1(+/+)-CDC20 (n=15)
Mlh1(+/+)-CDC20R132A (n=21)
Mlh1(-/-)-CDC20R132A (n=14)
40
30
20
10
0
M=20
M=20
M=15
p<0.001
p<0.001
M=5.5
M=0
M=0
dyads
single chromatids

Figure S7

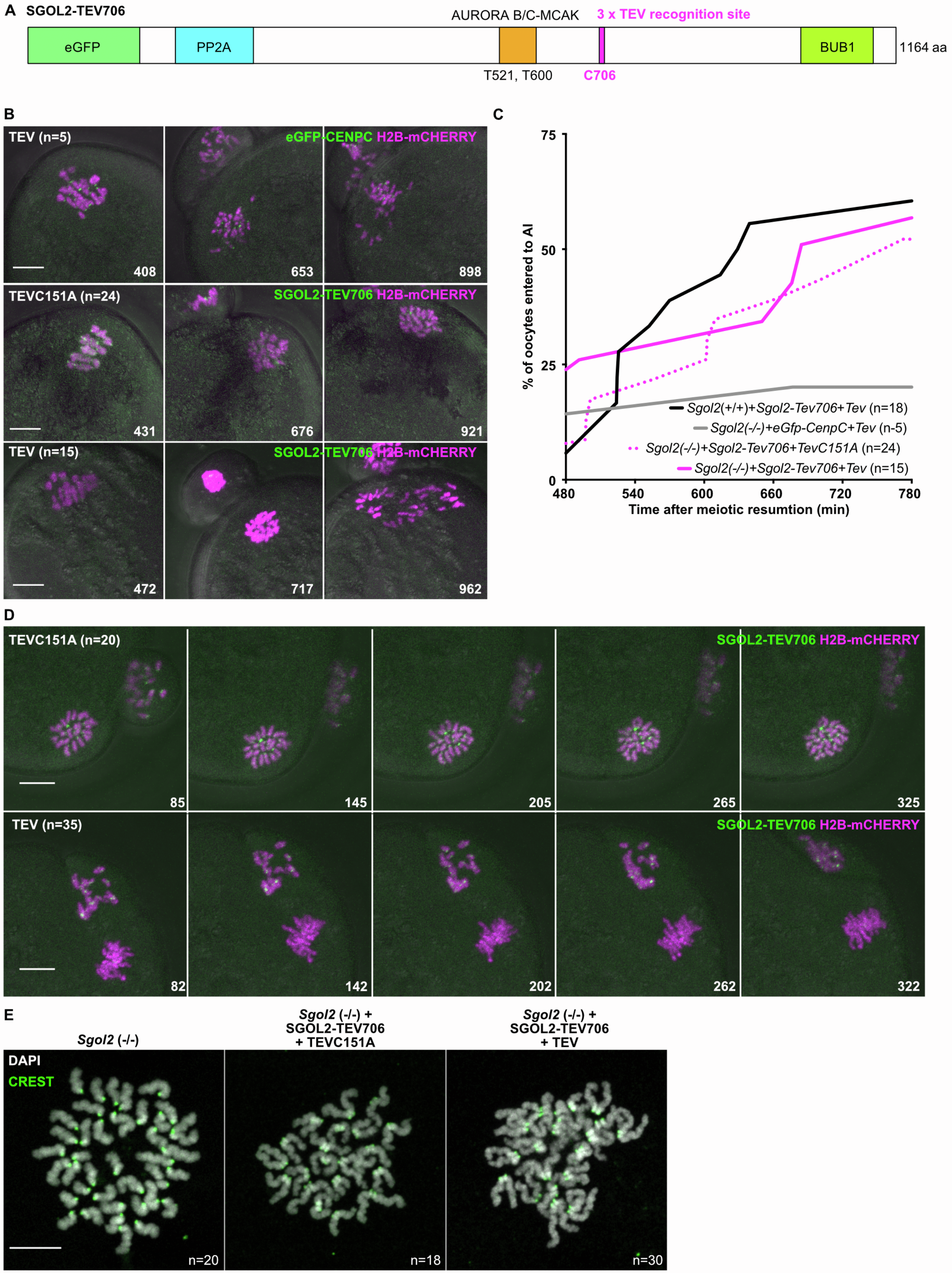
A SGOL2-TEV706
AURORA B/C-MCAK
3 x TEV recognition site
eGFP
PP2A
BUB1
1164 aa
T521, T600
C706
B
TEV (n=5)
eGFP-CENPC H2B-mCHERRY
408
653
898
TEVC151A (n=24)
SGOL2-TEV706 H2B-mCHERRY
431
676
921
TEV (n=15)
SGOL2-TEV706 H2B-mCHERRY
472
717
962
C
% of oocytes entered to AI
Time after meiotic resumtion (min)
Sgol2(+/+)+Sgol2-Tev706+Tev (n=18)
Sgol2(-/-)+eGfp-CenpC+Tev (n-5)
Sgol2(-/-)+Sgol2-Tev706+TevC151A (n=24)
Sgol2(-/-)+Sgol2-Tev706+Tev (n=15)
D
TEVC151A (n=20)
SGOL2-TEV706 H2B-mCHERRY
85
145
205
265
325
TEV (n=35)
SGOL2-TEV706 H2B-mCHERRY
82
142
202
262
322
E
Sgol2 (-/-)
Sgol2 (-/-) + SGOL2-TEV706 + TEVC151A
Sgol2 (-/-) + SGOL2-TEV706 + TEV
DAPI
CREST
n=20
n=18
n=30

Figure S1 Either Sister Kinetochore Bi-orientation or De-protection of Cohesion Is Conferred by the Nature of Dyads Itself rather than the Surrounding Factors Coming from Cytoplasm, related to Figure 1

(A) Live cell imaging of dyad segregation in an MI oocyte without fusion procedure (MI) and in that fused with an MII cytoplast (MIIcyt+MI). H2B-mCHERRY (magenta), chromosomes. eGFP-CENPC (green), kinetochores (KTs). Numbers indicate the time after meiotic resumption (min). Bars, 10 μm.

(B) Spindle disassembly of SCC containing dyads from MII fused with an oocyte at the germinal vesicle (GV) stage (MII-SCC+GV, (TUBULIN: green; DAPI: grey). Dashed lines indicate transplanted dyads. Numbers indicate the time after fusion. h, hour, Bar, 10 μm.

(C) Timing of dyad segregation in MII-SCC+GV. As a technical control, MII cytoplasm was fused with a GV oocyte (MIIcyt+GV). Log-rank test was performed, but there was no significant difference ($p<0.01$). n, the numbers of oocytes measured.

(D) Live cell imaging of dyad segregation in MII-SCC+GV as in Figure S1A. Bars, 10 μm.

(E) Percentage of oocytes extruded polar bodies (Pbs) without doing live cell imaging. One-way ANOVA followed by Tukey's multiple comparison test was performed, but there was no significant difference ($p<0.01$).

(F) Live cell imaging showing that dyad biorientation in MII-SCC+GV as in Figure S1A. Arrow heads indicate bi-orientated dyads and arrows indicate dyads failed to associate with a spindle. Bar, 10 μm.

(G) Percentage of oocytes activated 1 hour after artificial strontium activation. One-way ANOVA followed by Tukey's multiple comparison test was performed, but there was no significant difference ($p<0.01$).

n, the numbers of oocytes analysed.

Figure S2 Determination of Protection of Cohesion or Sister Kinetochore Co-orientation Is Granted by Bivalents rather than the Factors from Cytoplasm, related to Figure2

Live cell imaging of bivalent segregation in an MII oocyte without fusion procedure (MII) and an MII oocyte fused with an MI cytoplast (MIcyt+MII) as in Figure S1A. Bars, 10μm. n, the numbers of oocytes analysed.

Figure S3 Dyads that Have Undergone Two Successive Meiotic Divisions Are Converted to Single Chromatids after Artificial Strontium Activation, related to Figure 3

(A) The comparable amount of REC8 was loaded on bivalents from a *Zp3Cre Separase* (f/f) oocytes (*Sep* (-/-)) at MI, whose meiotic arrest defects were rescued by expression of Separase at the GV stage (*Sep* (-/-)+SEP), and *Sep* (-/-) oocytes at MII induced only cytokinesis by expression of catalytic-dead version of Separase C2028S (*Sep* (-/-) C2028S). The antibodies shown in the panels (green and magenta). DNA was stained with DAPI (grey). Bars, 10μm.

(B) Segregation timing after artificial strontium activation in dyads from *Sep* (-/-) oocytes. *Sep* (-/-)+SEP-SCC+MIcyt: an SCC from a *Sep* (-/-)+SEP oocyte was fused with an MI cytoplast from wild-type, *Sep* (-/-) C2028S-SCC+MIcyt: an SCC from a *Sep* (-/-) oocyte induced only cytokinesis by expression of catalytic-dead version of Separase C2028S was fused with an MI cytoplast from

wild-type. In both groups, dyads were prepared by induction of MI-MII transition after fusion. Log-rank test was performed, but there was no significant difference ($p<0.01$).

(C) Live cell imaging showing that dyads that have successfully undergone three successive divisions after artificial activation as in Figure S1A. Numbers indicate the time after artificial activation (min). *Sep* (-/-)+SEP: *Sep* (-/-)+SEP-SCC+MIcyt, *Sep* (-/-)+C2028S: *Sep* (-/-)+C2028S-SCC+MIcyt. Bars, 10 μm.

(D) Formation of single chromatids from dyads that have undergone three successive divisions after artificial activation (CREST: green; DAPI: grey). Bar 15 μm.

n, the numbers of oocytes analysed.

Figure S4 REC8 Localization at Centromeric, Peri-centromeric and Arm Regions in Bivalents (MI), and at the Distal Side of Peri-centromeric Regions in Dyads (MII), related to Figure 4

(A) Centromeric regions were marked by CREST and peri-centromeric regions were marked by histone H3 tri-methylated at lysine 9 (H3K9me3) (Top) or topoisomerase II (TOPOII) (Bottom). The antibodies are shown in the panels (yellow, cyan and magenta). DNA was stained with DAPI (grey). Bars, 5 μm.

(B) The axis length of peri-centromeric regions in mouse oocytes at MI. White circle, median (M).

n, the numbers of oocytes/peri-centromeric regions analysed.

Figure S5 Disappearance of Centromeric REC8 Does Not Alter Localization of SGOL2 at MI, related to Figure 7

Left, Schematic of experiments showing that the induction of specific cleavage of centromeric REC8 by CCTEV. Right, A representative 3D-SIM image showing that retained localization of SGOL2 and Meikin at centromeric regions 6 hours after induction of CCTEV expression. CCTEVC151A is a catalytic dead version of CCTEV. Insets show 1.5-fold magnification of the regions indicated in the dash-lined boxes. The antibodies are shown in the panel (green and magenta). DNA was stained with DAPI (blue). Bars, 5 μm. n, the numbers of oocytes analysed.

Figure S6 Loss of Co-orientation of Sister Kinetochores Triggers Deprotection of Peri-centromeric REC8 during MI-MII Transition, related to Figure 7

(A) A representative 3D-SIM image showing bi-orientation of sister kinetochores in two univalents (arrow heads) from *Mlh1* (-/-) oocytes. The antibodies are shown in the panel (green and magenta). DNA was stained with DAPI (blue). Bar 5μm.

(B) Segregation timing of bivalents/univalents after expression of CDC20R132A in an MI oocyte. Oocytes expressed with CDC20R132A underwent the first meiotic division with an average timing of 321±83 min (14/18), which is substantially faster than those expressed with CDC20 (5/8, 605±119 min). Log-rank test was performed to test significance and only non-significance (ns) was shown ($p<0.01$).

(C) Schematic of experiments (left) and representative stills (right) showing the induction of first meiotic division by inhibition of the spindle check point using expression of CDC20R132A in *Mlh1*(-/-) oocytes. H2B-mCHERRY (magenta), chromosomes. SECURIN-eGFP (green), whose

destruction indicates first anaphase entry and re-accumulation shows entry to meiosis II. Bars, 10 μm.

(D) A representative image of chromosome spread showing formation of dyads from univalent (CREST: green; DAPI: grey). Bar, 10 μm.

(E) Number of dyad or single chromatid formation after the first meiotic division in *Mlh1*(-/-) oocytes injected with *CDC20R132A* mRNA. Boxes show the median, 25th and 75th percentiles, and bars show the 10th and 90th percentiles. One-way ANOVA followed by Tukey's multiple comparison test was performed (p<0.01). M, Median.

n, the numbers of oocytes analysed.

Figure S7 SGOL2 in meiosis II Is Not Required for Protection of Cohesin in Dyads, related to Figure 7

(A) Insertion site of 3xTEV-recognition sequence in *Mus musculus* SGOL2 at cysteine 706. Each colored box shows a binding region of indicated protein or conjugated eGFP at N terminus.

(B) Live cell imaging showing that the defect of single chromatid formation in *Sgol2 (-/-)* oocytes was rescued by expression of TEV-cleavable SGOL2, SGOL2-TEV706 (green, middle), but not by expression of eGFP-CENPC (green, top). Expression of TEV at MI induced single-chromatid formation in *Sgol2 (-/-)* oocytes rescued by expression of SGOL2-TEV706 (green, bottom). Labels on each left top panel indicate a type of injected *Tev* mRNA at MI. Numbers indicate the time after meiotic resumption (min). H2B-mCHERRY (magenta), chromosomes. TEVC151A, a catalytic dead version of CCTEV. Bars, 10 μm.

(C) Segregation timing of bivalents in *Sgol2 (-/-)* oocytes after expression of SGOL2-TEV706. After *Sgol2-Tev706* mRNA injection at the GV stage, chromosome segregation occurred at the average timing of 602±96 min and 595±118 min in *Sgol2 (+/+)* and *Sgol2 (-/-)* oocytes, respectively; 75% (58/77) of *Sgol2 (+/+)* and 76% (102/134) of *Sgol2 (-/-)* oocytes were extruded a polar body. All bivalents in *Sgol2 (-/-)* oocytes that had injected *Sgol2-Tev706* mRNA (300 ng/μl) at the GV stage and *TEVC151A* mRNA (300 ng/μl) at MI, respectively, segregated to dyads at the rate of 50% (12/24). In contrast, the frequency of chromosome segregation in *Sgol2 (-/-)* oocytes that had injected *Sgol2-Tev706* mRNA at the GV stage and, subsequently, *TEV* mRNA (300 ng/μl) at MI, was at 47% (7/15) and all bivalents from these oocytes segregated to single chromatids. Numbers indicate the time after meiotic resumption (min). Log-rank test was performed to test significance, but there was no significant difference (p<0.01).

(D) Live cell imaging showing the maintenance of dyads even after cleavage of SGOL2-TEV706 (green) at MII. Labels on each left top panel indicate a type of injected *Tev* mRNA (300 ng/μl) at MII. Numbers indicate the time after induction of TEV expression (min). H2B-mCHERRY (magenta), chromosomes. Bars, 10 μm.

(E) Maintenance of dyads even after destruction of SGOL2 function at MII (CREST: green; DAPI: grey). Bar, 10 μm.

n, the numbers of oocytes analysed.
